# Rubrene–Oxalate Electrochemiluminescence Reveals Physicochemical Properties of Triphasic Boundaries

**DOI:** 10.1021/cbmi.5c00295

**Published:** 2026-04-03

**Authors:** Megan L. Hill, Mackenzie P. Stockwell, Jeffrey E. Dick

**Affiliations:** Department of Chemistry, 311308 Purdue University, West Lafayette, Indiana 47907, United States

**Keywords:** rubrene, oxalate, electrochemiluminescence, triple-phase boundary, microdroplet, liquid|liquid|electrode interface, dynamic movement

## Abstract

Multiphase chemical systems are abundant in nature and widespread in industrial and pharmaceutical settings. Moreover, multiphase, microconfined systems have been implicated in unique and fascinating chemistries, such as reaction acceleration, unexpected chemical transformations, and reactive species production. Despite the importance and prevalence of multiphase environments, they remain challenging to study. Hence, alternative strategies are necessary to investigate these systems. In this study, we present an electrochemiluminescence (ECL) system that selectively illuminates triphasic boundaries (liquid|liquid|electrode interfaces) within multiphase systems. This triphasic boundary-confined ECL was achieved using rubrene as the luminophore, which is only soluble in organic solvents, and oxalate as the coreactant, whose solubility is limited to the aqueous phase. With this imaging platform, we interrogated single microdroplets, microdroplet arrays, and a bulk liquid|liquid|electrode interface. By restricting the ECL signal to the triphasic boundaries present in each of these systems, we mapped these boundaries within microdroplet arrays and tracked and imaged the dynamic movement of droplets, specifically a reorganization event and a coalescence event, in real time. Moreover, this system revealed differences in a 3D space by showing distinct ECL emission layer diffusion patterns at two unique imaging planes of a bulk liquid|liquid|electrode interface. Our results demonstrate ECL microscopy’s ability to interrogate complex, multiphase systems in real time.

## Graphical abstract

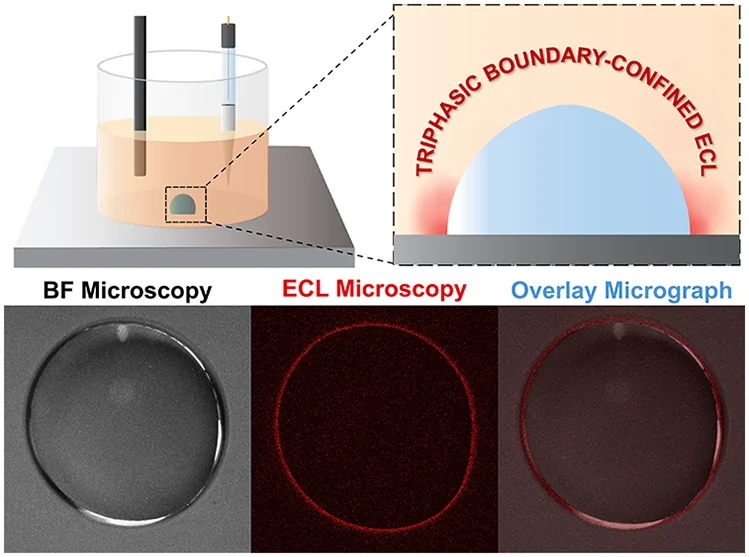

## Introduction

Multiphase environments are ubiquitous in nature and can be found within biological systems, such as nonmembranous cell organelles (e.g., ribosomes),^1^ and the atmosphere (e.g., clouds).^2^ Moreover, these multiphase systems have gained increasing interest within industrial and pharmaceutical settings, often being used in “on-water” organic catalysis reactions.^3–5^ Specifically, emulsions, mixtures composed of two immiscible liquids (e.g., oil and water),^6,7^ have been employed in areas such as materials synthesis and drug development.^8–12^ Notably, within the past couple of decades, multiphase, microconfined systems have been implicated in curious chemistries.^13–18^ Largely, these chemistries have been observed in microdroplets, which exhibit properties such as reaction acceleration,^19–21^ unexpected chemical transformations,^22–25^ and the production of highly reactive species.^26–29^ Despite the prevalence and versatility of multiphase environments, many aspects of these systems remain elusive. For instance, the physical underpinnings driving the unique properties of multiphase, microconfined systems remain a subject of ongoing discussion.^30–37^ Furthermore, dynamic processes at droplet|bulk|electrode interfaces, such as droplet reorganization events (coalescence, splitting, wetting, etc.), variances in interfacial thickness, and phase- and charge-transfer processes, continue to be enigmatic.^38–42^ Considering the aforementioned knowledge gaps, further investigations of multiphase systems, especially on electrified surfaces, are warranted. To develop a complete conceptual framework for these complex and difficult-to-study environments, new methodologies and innovative studies are necessary.

Recently, electrochemiluminescence (ECL) microscopy has emerged as a promising and effective technique to examine multiphase environments.^43^ ECL microscopy is a technique that uses redox reactions at the surface of an electrode to promote a luminophore to an excited state.^44–46^ This excitation can occur either in the presence or absence of a sacrificial coreactant.^47^ The high-energy luminophore species then emits light upon relaxation to the ground state, subsequently producing an optical signal. The dependency of the ECL emission layer on the short lifetimes of the radical intermediates (often microseconds) enables this technique to have distinct and unique advantages.^48,49^ Foremost, ECL microscopy has excellent spatiotemporal resolution and near-zero background noise, making it a premier technique for interrogating surface-confined processes.^44,46^ Previously, ECL microscopy has visualized dynamic, multiphase environments.^50–57^ Our group has interrogated multiphase systems that include entrapped solvents within droplets^58^ and the formation of abiotic microcompartments upon droplet coalescence.^59^ Separately, Sojic and co-workers have imaged the propulsion of electrogenerated bubbles into a solution^60^ and have conducted bipolar ECL at the aqueous|organic|microbead electrode interface.^61^ In these investigations, typically, the ECL emission is limited to one of the phases (aqueous or organic) to selectively study that phase. In some cases, multicolor ECL is employed to simultaneously, or near-simultaneously, interrogate both phases.^53^

Triphasic boundaries, specifically gas|liquid|electrode and droplet|bulk|electrode interfaces, have been previously investigated by our group.^62^ In that work, we isolated ECL emission to the triphasic boundary of an aqueous droplet settled on an electrode and within the organic bulk phase. We achieved this by loading our aqueous droplet with sodium oxalate and including Ru­[(dmbpy)_3_]­(PF_6_)_2_ (an organic-soluble, methylated [Ru­(bpy)_3_]^2+^ derivative) in our bulk organic phase. The current work expands on this by using rubrene, a polycyclic aromatic hydrocarbon species that is highly hydrophobic, as the organic phase luminophore.^54^ Rubrene is particularly advantageous for this purpose because it is exceedingly reactive with water and oxygen (unlike methylated [Ru­(bpy)_3_]^2+^ derivatives), preventing it from entering the aqueous phase.^63^ Furthermore, polyaromatics are found extensively throughout the petroleum industry, and it is important to understand their reactivity at complex phase boundaries that may occur during oil spills.^64–67^ In this study, we probe various liquid|liquid|electrode triphasic boundaries using the ECL system of a rubrene luminophore (organic phase) and a sodium oxalate coreactant (aqueous phase). By restricting the ECL signal to the triphasic boundary, we reveal insights into the dynamic movement of droplets on an electrode, identify droplet–bulk–electrode boundaries within a microdroplet array, and demonstrate ECL emission layer morphology differences when interrogating bulk interfaces. Herein, we present a new, triphasic boundary-confined imaging platform that may lay the foundation for future ECL microscopy investigations of complex multiphase systems.

## Results and Discussion

In the first set of ECL microscopy experiments, we carefully pipetted a ca. 2 μL aqueous microdroplet loaded with 100 mM sodium oxalate (Na_2_C_2_O_4_) and 100 mM potassium chloride (KCl) onto an optically transparent, indium tin-doped oxide (ITO) electrode within an opto-electrochemical cell. We then immersed the aqueous microdroplet in an organic bulk phase of 6 mM rubrene and 100 mM tetrabutylammonium perchlorate (TBAP) within 1,2-dicholorethane (1,2-DCE). Figure 1a shows this experimental setup, wherein the orange solution represents the organic bulk phase, while the blue droplet represents the aqueous microdroplet. We employed a three-electrode electrochemical cell for these experiments, where ITO is the working electrode (bottom of the opto-electrochemical cell), a glassy carbon (GC) rod is the counter electrode (left electrode), and an Ag/AgCl/1 M KCl electrode, connected to the system via an agarose salt bridge, is the reference electrode (right electrode). To carry out the ECL microscopy, we placed the opto-electrochemical cell above the inverted optical microscope, which was modified to include both an EMCCD camera (for ECL microscopy) and a CMOS camera (for brightfield (BF) imaging). Figure S1 shows the full experimental setup. Further experimental information is detailed in the Materials and Methods section of the Supporting Information.

**Figure 1:**
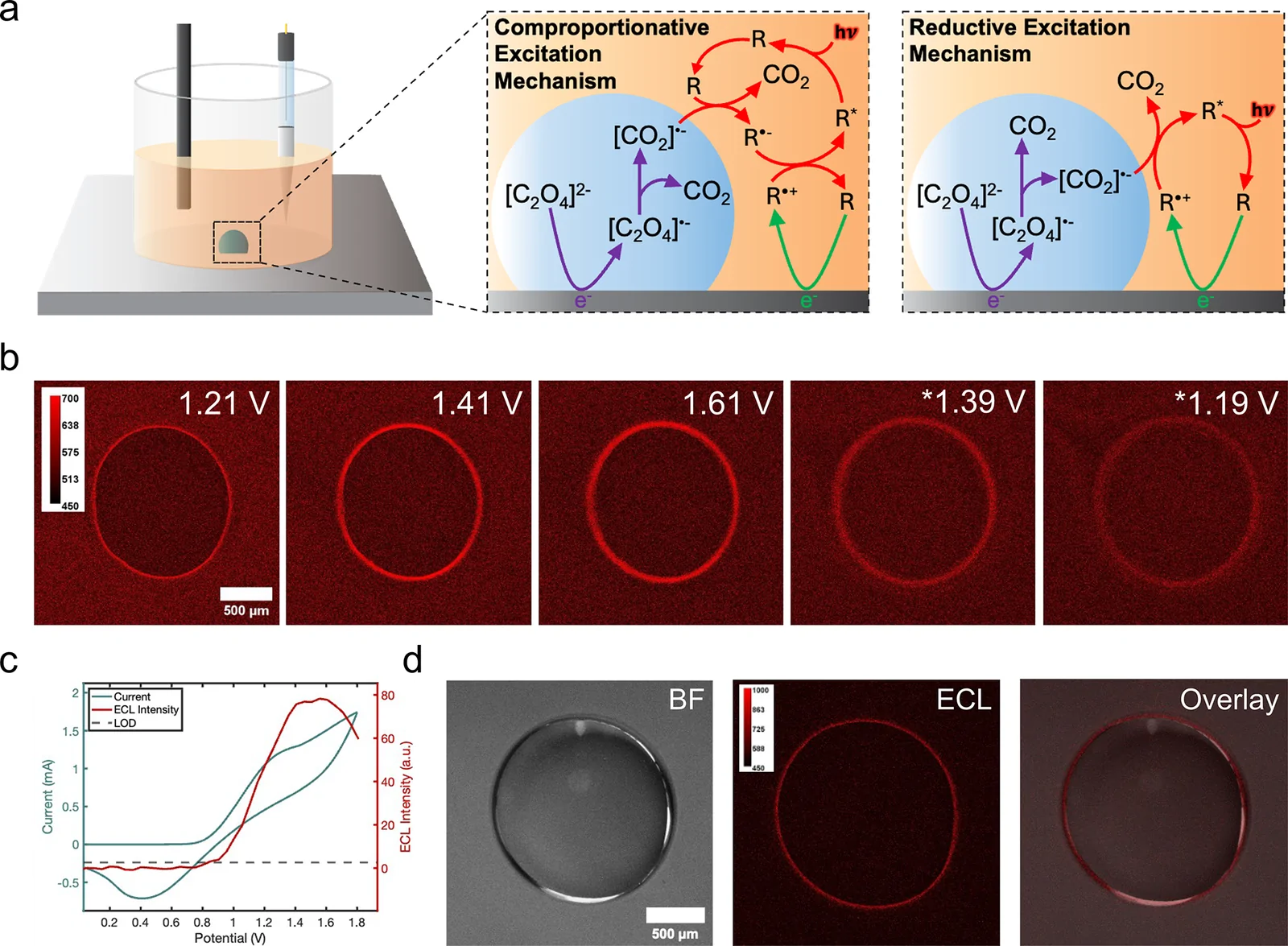
(a) Schematic of the experimental setup (far left) with two proposed mechanisms for the ECL reaction. The two proposed mechanisms are a comproportionative excitation mechanism (center) and a reductive excitation mechanism (right). (b) Series of ECL micrographs demonstrating how the ECL emission layer changes over the course of a cyclic voltammogram (CV). The potential window for the CV was 0–1.8 V vs Ag/AgCl/1 M KCl, and the scan rate was 50 mV/s. The * symbol indicates that the indicated potential was on the backsweep of the CV. The system consisted of a ca. 2 μL aqueous droplet composed of 100 mM sodium oxalate and 100 mM KCl on an ITO working electrode. The droplet was immersed in a bulk organic phase of 6 mM rubrene and 100 mM TBAP within 1,2-DCE. (c) Correlated ECL intensity and cyclic voltammetry plot. The dashed line shows the limit of detection (LOD) for the EMCCD camera (detailed in the Materials and Methods section of the Supporting Information). The CV was taken in a potential window of 0–1.8 V vs Ag/AgCl/1 M KCl at a scan rate of 50 mV/s. The system was composed of an approximately 2 μL aqueous droplet with 100 mM sodium oxalate and 100 mM KCl on an ITO working electrode. The droplet was immersed in a bulk organic phase of 10 mM rubrene and 100 mM TBAP within 1,2-DCE. (d) Brightfield (BF) micrograph (left), ECL micrograph (right), and overlay of the BF and ECL micrographs. The BF micrograph was taken with a CMOS camera. The experimental setup was identical to the setup described for (c). All scale bars correspond to 500 μm.

To achieve triphasic boundary-confined ECL emission, we devised an ECL system wherein the luminophore and the coreactant are soluble in separate phases (i.e., organic vs aqueous phases). Rubrene was the ideal candidate for the luminophore because it is readily soluble in many common organic solvents, including 1,2-DCE, and it is highly hydrophobic and reactive with oxygen and has poor radical stability in water.^54,63^ Hence, rubrene is restricted to the organic phase with negligible leeching into the aqueous phase. Moreover, the poor rubrene radical stability in water prevents ECL emission from the aqueous phase, making it particularly advantageous over other water-insoluble luminophores such as Ru­[(dmbpy)_3_]­(PF_6_)_2_. Additionally, we chose rubrene as the luminophore because it is a representative, high-ECL efficiency, and relatively inexpensive polycyclic aromatic hydrocarbon (PAH). PAHs are of interest due to their unique optical and electronic properties^63^ as well as for their importance in the petroleum industry.^64–67^ For the coreactant, we selected sodium oxalate as it is insoluble in the 1,2-DCE phase but can easily dissolve into aqueous solutions at high (100 mM) concentrations.^56^ We hypothesized that given that (1) an applied potential is necessary to drive the ECL reactions and (2) the only place in the system where the luminophore and the coreactant meet is at the droplet|bulk interface, illumination should only occur at the triphasic (aqueous droplet|organic bulk|electrode) boundary. Notably, there is literature precedent for this proposed rubrene–oxalate ECL system. In 1977, Bard and co-workers demonstrated that a weak ECL emission is detectable upon the simultaneous oxidation of rubrene and oxalate dissolved within *N*,*N*-dimethylformamide (DMF) and, separately, a 1:1 benzene (BZ):acetonitrile (ACN) mixture.^68^ It is important to highlight here that a significant advantage of the presented rubrene/oxalate ECL system is the restriction of the rubrene and oxalate to separate phases, which, to the authors’ knowledge, has not been previously investigated.

We first tested the posited rubrene/oxalate triphasic boundary-confined ECL system by performing CV in the potential window of 0 to 1.8 V vs Ag/AgCl/1 M KCl at 50 mV/s. We did this to both validate Bard et al.’s findings that an anodic potential is necessary for the emission of light in this reaction^68^ and to examine the behavior of the ECL emission layer over the application of different anodic potentials. This trial is shown in Figure 1b, where the ECL emission appears to be the most intense at and primarily limited to the droplet|bulk|electrode interface, thereby supporting the hypothesized triphasic boundary-confined nature of this ECL system. The CV that corresponds to Figure 1b is shown in Figure S2. As depicted in Figure 1b, we observed the first bright frame of ECL emission at approximately 1.2 V. Interestingly, in this micrograph, resolvable ECL emission occurs in the bulk organic phase, indicating that some oxalate must leave the aqueous droplet. The phase transfer of the oxalate anion into the organic, bulk phase may be facilitated by the organic supporting electrolyte cation, TBA^+^, a well-known phase transfer agent, with electroneutrality maintained from the movement of perchlorate anions from the organic bulk into the aqueous droplet.^7^ Figure S3 illustrates this proposed mechanism, and Figure S4 presents a series of micrographs from various trials where other organic supporting electrolytes were examined. We learned that changing the anion of the organic supporting electrolyte (comparing Br^–^, Cl^–^, PF_6_^–^, and ClO_4_^–^) impacts the ECL intensity within the bulk organic phase.

Moreover, in Figure 1b, it is important to highlight how the first few frames of ECL emission have crisp, bright ECL emission. However, as the applied anodic potential increases, the width of the ECL emission layer continues to increase as well, as shown in the ECL micrographs at ∼1.4 and 1.6 V. Whereas, once the backsweep of the cyclic voltammogram (CV) is reached, not only does the ECL emission layer continue to increase in thickness, but also the emission intensity drops significantly. This is seen in the ECL micrographs at ca. 1.4 V and ca. 1.2 V of the CV backsweep, where the ECL intensity is much lower than in the earlier micrographs. The depletion of rubrene and/or oxalate at the triphasic boundary faster than their diffusion to the boundary likely contributes to the decrease in the ECL intensity. We support this hypothesis with the correlated current–ECL intensity–applied potential plot shown in Figure 1c. In this plot, it is shown that the ECL emission intensity is maximized between the potentials of 1.4 and 1.6 V before dropping once the applied potential continues past 1.6 V. Furthermore, we postulate that the observed increase in the ECL emission layer thickness during the CV is due, at least in part, to the diffusion of excited-state species or radical intermediates of the ECL reaction away from the triphasic boundary over time.^69^ Consequently, these results imply that only the first few frames of the ECL emission of this system should be considered when using this system to accurately map the location of triphasic boundaries. Also, it is important to note that the ECL emission is, overall, dim and requires the use of an EMCCD camera with at least 1 s of exposure and substantial electron multiplier gain. This dimness may be caused by the aforementioned reactivity of rubrene and rubrene radicals with water and oxygen and is a limitation of the system.

To present a complete conceptual framework of this rubrene/oxalate system and to hypothesize what contributes to the described “widening” of the ECL emission layer, it is critical to discuss potential ECL mechanisms for this system. In Figure 1a, we present two mechanisms: a comproportionative excitation mechanism (left dashed box) and a reductive excitation mechanism (right dashed box).^47^ In the first step of both posited reactions, simultaneously, an oxalate anion ([C_2_O_4_]^2–^) and a rubrene molecule (R) are oxidized. It should be emphasized that the simultaneous oxidation of rubrene and oxalate occurs only at applied potentials of 1.2 V vs Ag/AgCl and above when on an ITO working electrode. As shown in Figure S5, rubrene oxidation begins at ca. 0.8 V, while oxalate oxidation starts at approximately 1.2 V. Rubrene oxidizes into a rubrene radical cation (R^•+^), as represented in the reaction with the green arrow and electron in both mechanism schematics.^54,63^ The oxidation of the oxalate anion (purple arrows) results in the formation of an oxalate radical anion ([C_2_O_4_]^•‑^) that quickly decomposes into a carbon dioxide anion radical ([CO_2_]^•‑^) and a carbon dioxide gas molecule (CO_2_).^52,68,70^ Following this step, the mechanisms of the synthesis deviate. In the comproportionative excitation reaction (specified with the red arrows in the left schematic of Figure 1a), the [CO_2_]^•‑^ reduces rubrene to form a rubrene radical anion (R^•‑^) and CO_2_. Then, in a radical annihilation reaction, the R^•‑^ and R^•+^ annihilate to form excited-state rubrene, R*, and ground-state R. In these proposed mechanisms, rubrene is recycled, while oxalate, a sacrificial coreactant, decomposes during the reaction. Once R* is formed, it subsequently releases a photon at either 564 or 595 nm, as the previously reported ECL spectrum has two wavelength maxima,^54,71^ when it relaxes back to the ground state.

Alternatively, in the reductive excitation reaction (specified with the red arrows in the right schematic of Figure 1a), [CO_2_]^•‑^ directly reduces R^•+^ to form CO_2_ and the excited-state rubrene, R*. As described previously, light is emitted when the R* decays to R. To further validate these proposed mechanisms, we carried out ECL microscopy experiments without the presence of R and, separately, [C_2_O_4_]^2–^ in the system (Figures S6 and S7, respectively). These controls demonstrate that both R and [C_2_O_4_]^2–^ must be in the system for ECL emission to occur. Separately, Bard et al. hypothesized that, in the mechanism for this rubrene/oxalate anodic ECL system, the R^•+^ could oxidize [C_2_O_4_]^2–^ to form R and [C_2_O_4_]^•‑^.^68^ After this step, Bard et al. then proposed that the ECL reaction would follow the comproportionative excitation reaction mechanism. This posited mechanism by Bard is likely the dominant mechanism in the potential window of 0.8 to 1.2 V, as rubrene electrooxidation occurs at 0.8 V, yet oxalate electrooxidation does not occur until 1.2 V, as demonstrated in Figure S5. However, at potentials of 1.2 V and higher, this mechanism is not likely a primary contributing mechanism because the oxalate concentration present at the triphasic boundary is in over 10x excess as compared to the rubrene concentration (100 vs 10 mM, respectively). Hence, most R^•+^ would likely react with any [CO_2_]^•‑^ or R^•‑^ present. However, if some R^•+^ can diffuse away from the electrode surface but remain at the droplet/bulk interface, ECL emission may occur via this proposed mechanism. Moreover, this mechanism is plausible for the aforementioned “widening” of the ECL emission layer. Given that this “widening” only appears to occur in later ECL micrographs, this mechanism could become more dominant if the oxalate at the triphasic boundary becomes depleted, as less [CO_2_]^•‑^ may be present to react with R^•+^, allowing it to diffuse away from the boundary.

To further examine the effectiveness of this ECL imaging platform to selectively illuminate the triphasic boundary, we performed an experiment that is shown in Figure 1d. First, we took a brightfield micrograph of the aqueous droplet as seen in the micrograph labeled “BF”. Then, we carried out ECL microscopy, with the brightest of the first couple of ECL micrographs we collected during the experiment shown in the micrograph labeled “ECL”. After that, we overlaid the BF and ECL micrographs on each other, as shown in the micrograph labeled “Overlay”. As seen in this “Overlay” micrograph, the spatial region of the ECL emission closely matches the area in the BF micrograph where the edges of the droplet appear to be in contact with the electrode. These results further support that this rubrene/oxalate ECL system robustly elucidates droplet|bulk|electrode interfaces via its triphasic boundary-confined nature.

As we demonstrated that this rubrene/oxalate ECL system can selectively illuminate triphasic boundaries, the next important avenue of inquiry is whether this system can capture dynamic triphasic boundary movement. To probe this question, we performed an experiment where, using the same experimental setup described in Figures 1a and S1, we conducted CV to drive dynamic droplet movement. In the dashed ECL micrographs of Figure 2b, a droplet reorganization event is seen, wherein it appears that electrowetting of the droplet occurred, resulting in a change of contact area. This could be an example of voltage-induced electrowetting, which is a phenomenon wherein a liquid droplet on an electrified surface changes its contact angle due to the application of potential. Specifically, this occurs because when potential is applied, electrical charges accumulate at the droplet|electrode interface, altering the surface tension and causing the droplet to spread out, resulting in a decrease in the contact angle and increase in the contact area.^72^ Besides electrowetting, other possible driving forces for the droplet reorganization event could be electrolysis-originated surface tension gradients and electrolysis-driven density changes, which could be caused by Marangoni-driven convection at the insulator|electrode|electrolyte boundary.^73^ To explore this further, we tested different droplet volumes, shown in Figure S8, and we did not observe any droplet reorganization for droplets smaller than 2 μL. We include the corresponding CV to Figure 2b,2c in Figure S9. Figure 2a schematically depicts this droplet reorganization phenomenon. At time 0.00 s, the droplet is stable on the electrode surface. However, in less than a second, the droplet moves, as we depict with the central, distorted droplet in the schematic. The result of this event is a droplet with a larger contact area on the electrode. Figure 2c highlights the droplets before and after the reorganization event. In the ECL micrograph to the left, framed with a red border, the droplet, less than a second before the event, is shown, with a diameter of 1.128 mm. We measured this diameter based on the location of the triphasic boundary, which the ECL emission layer location reveals. In comparison, the ECL micrograph in the center, framed by a blue border, depicts the droplet immediately after the reorganization event. The time between the ECL micrograph on the left, before the event, and the one in the center, the first micrograph with the reorganized droplet, was ca. 1.00 s. This relatively short time frame between ECL micrographs highlights the quick nature of the reorganization event. Following the event, the diameter of the droplet was measured to be 1.381 mm; hence, the diameter increased by approximately 253 μm.

**Figure 2:**
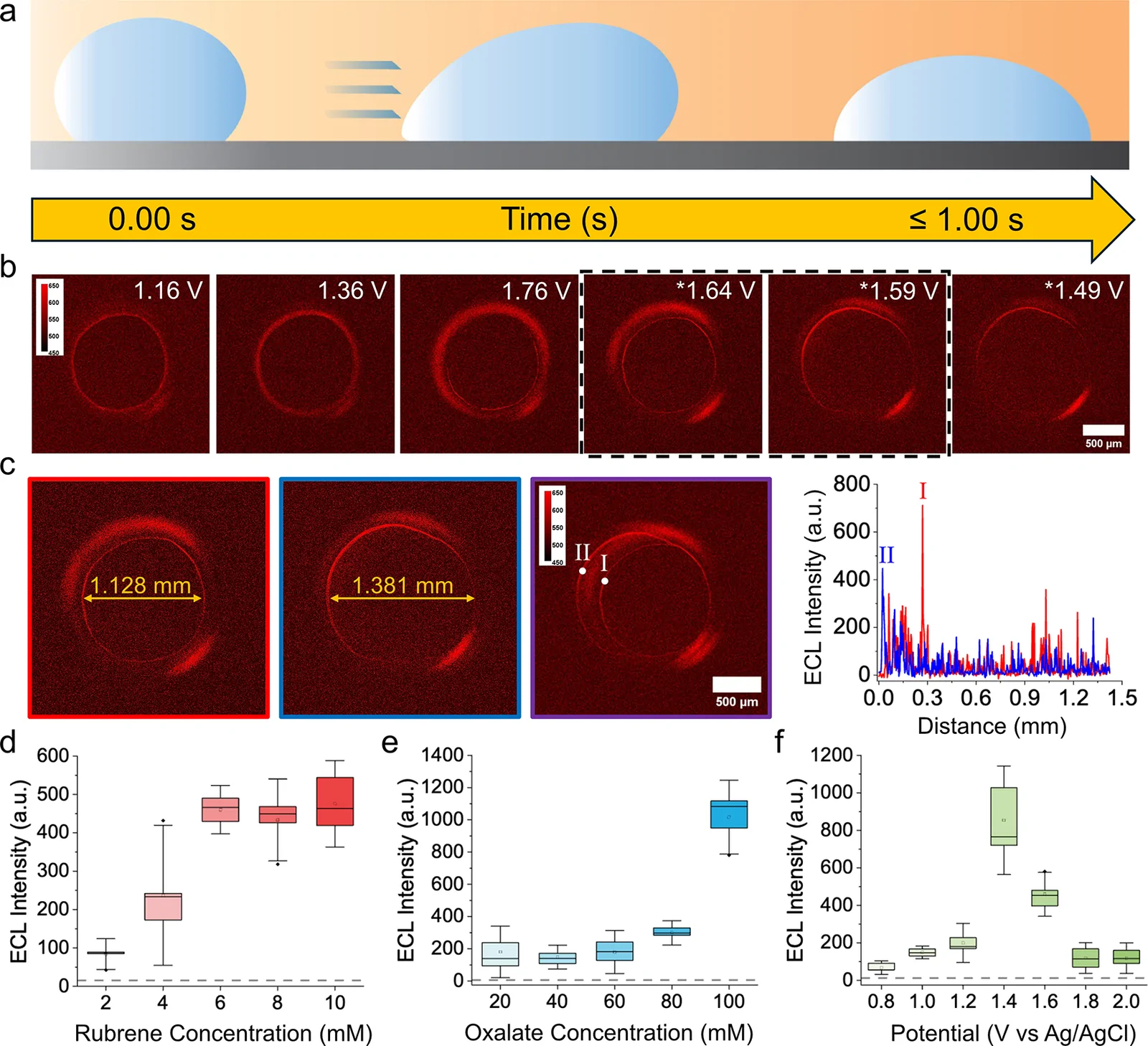
(a) Schematic depicting the droplet reorganization event over time. Initially, an aqueous droplet is settled on an ITO electrode, and then, in less than a second, the droplet moves and its contact area changes, leaving behind a reorganized droplet. (b) Series of ECL micrographs from the same experiment that include the droplet reorganization event. The frames immediately before and after the event are included in the dashed box. The potential window for the CV was 0 to 1.8 V vs Ag/AgCl/1 M KCl, and the scan rate was 50 mV/s. The * symbol indicates that the potential was on the backsweep of the CV. ECL microscopy was performed in a system consisting of a ca. 2 μL aqueous droplet with 80 mM sodium oxalate and 100 mM KCl on an ITO working electrode immersed in a bulk organic phase of 10 mM rubrene and 100 mM TBAP within 1,2-DCE. (c) Enlarged view of the frames immediately before (left) and after (center) the droplet reorganization event with the measured width of the droplet. The rightmost ECL micrograph is an overlay of the micrographs to its left. All three micrographs are at the same scale and calibration bar shown in the overlay micrograph. On the far right, an ECL intensity vs distance plot, collected from a line scan, demonstrates the movement of the triphasic boundary because of the event. The symbols “I” and “II” on the overlay micrograph correspond to the “I” (red, before event) and “II” (blue, after event) on the plot, respectively. (d) Plot of ECL intensity versus rubrene concentration. For these experiments, ECL microscopy was performed on an approximately 2 μL aqueous droplet with 100 mM sodium oxalate and 100 mM KCl on an ITO working electrode immersed in a bulk phase of rubrene at varying concentrations (2, 4, 6, 8, and 10 mM) and 100 mM TBAP within 1,2-DCE. The potential window for the CV was 0 to 1.8 V vs Ag/AgCl/1 M KCl, and the scan rate was 50 mV/s. (e) Plot of ECL intensity vs oxalate concentration. For these experiments, ECL microscopy was conducted on an approximately 2 μL aqueous droplet with varying sodium oxalate concentrations (20, 40, 60, 80, and 100 mM) and 100 mM KCl on an ITO working electrode immersed in a bulk phase of 10 mM rubrene and 100 mM TBAP within 1,2-DCE. The potential window for the CV was 0 to 1.8 V vs Ag/AgCl/1 M KCl, and the scan rate was 50 mV/s. (f) Plot of ECL intensity versus applied potential. For these experiments, ECL microscopy was carried out in a system consisting of a ca. 2 μL aqueous droplet with 100 mM sodium oxalate and 100 mM KCl on an ITO working electrode immersed in a bulk organic phase of 10 mM rubrene and 100 mM TBAP within 1,2-DCE. To drive the ECL reaction, amperometric *i*–*t* curves held for 60 s at different applied potentials were used (0.8 1.0, 1.2, 1.4, 1.6, 1.8, and 2.0 V vs Ag/AgCl/1 M KCl). In the box-and-whisker plots shown in Figure 2d, e, and f, the central square represents the average ECL intensity, the middle horizontal line within the box represents the median ECL intensity, the colored portions of the box represent where ∼25 to 75% of the ECL intensity values lie, the bars (segmented vertical lines) represent 1.5 times the interquartile range of the ECL intensity values, and the diamonds outside of the bars represent outliers. The specific experimental details are also included in the Materials and Methods section of the Supporting Information. The dashed lines indicate the LOD values for the EMCCD camera used to collect the ECL micrographs. All scale bars correspond to 500 μm.

To more clearly demonstrate the occurrence of this droplet reorganization event, the ECL micrograph on the right in Figure 2c shows an overlay of the ECL micrographs that we collected immediately before and after the event (the two micrographs to the left of this micrograph). We also took line scans to further interrogate this reorganization event elucidated by the triphasic boundary-confined ECL. One line was drawn through the droplet after reorganization, so that each end of the line fell on either side of the ECL emission at the triphasic boundary. The same line was used for the line scan of the ECL micrograph right before and right after the reorganization event (shown in Figure S10). A plot of the ECL intensity versus distance on this line is shown in the far right of Figure 2c. Region I highlighted on the overlaid ECL micrograph corresponds to the “I” labeled on the plot. As seen in the plot, this region has a sharp ECL emission at ca. 270 μm on the red line. Similarly, Region II included on the overlaid ECL micrograph corresponds to the “II” labeled on the plot, which shows that this sharp ECL emission shifts by ca. 23 μm on the blue line following the reorganization event. Thus, the line scan further validates this observed shift in the triphasic boundary, as revealed by the ECL emission. Moreover, this exemplifies how this triphasic boundary-confined ECL system can not only visualize the dynamic movement of the triphasic boundary but also be used as a tool to track how the boundary changes over the course of an experiment.

Notably, the ECL micrographs shown in Figure 2b,2c show a heterogeneous “ring” of ECL emission that can be seen around the ECL signal from the triphasic boundary. This “ring” appears to be up to tens of micrometers away from the electrode surface, which extends the ECL emission layer much farther away from the electrode surface than the diffusion of rubrene and oxalate and the lifetimes of their intermediates would predict. This “ring” could be caused by optical effects such as lensing effects. Lensing effects have been documented in other ECL systems, typically occurring when the curvature of a droplet acts like a lens and focuses light to specific parts of the droplet.^57,74^ Separately, this “ring” could arise from the width of the liquid|liquid interface between the aqueous droplet and the organic bulk. Previous reports by our group and others have measured the liquid|liquid interfacial width under electrochemical conditions to be up to microns in size.^59,62,75,76^ If this is the case, then, the observed heterogeneous “ring” of ECL emission could be reflective of the liquid|liquid interface width between the droplet and the bulk solution. The ECL micrograph collected at 1.64 V in the backsweep of the CV in Figure 2b (which is also the leftmost ECL micrograph in Figure 2c) most clearly demonstrates this “ring” of ECL emission. In the ECL micrograph, the emission at the triphasic boundary is distinct, as evidenced by the thin but intense emission region. However, a diffuse, heterogeneous ECL emission region is also observed directly outside of the triphasic boundary-confined ECL signal. Interestingly, this diffuse ECL emission also changes with the triphasic boundary-confined signal, as seen in the ECL micrograph collected at 1.59 V in the backsweep of the CV in Figure 2b (which is also the central ECL micrograph in Figure 2c). The shift in the location of the triphasic boundary caused by the reorganization event consequently also appears to shift this diffuse region of ECL emission, where, in this later ECL micrograph, this diffuse signal is visible both partially inside and outside the triphasic boundary. This ECL emission can also be observed on the line scan plot (ECL intensity vs distance plot) on the far right of Figure 2c. In the line scan taken before the reorganization event (red line), between approximately 40 and 200 μm just to the left of the sharp ECL emission attributed to the triphasic boundary-confined ECL signal, there is an increased ECL intensity. This ECL intensity, which is a little less than half of the emission from the triphasic boundary-confined ECL, aligns with the direction in which the diffuse ECL emission is seen on the correlated ECL micrograph. It is important to note that this diffuse, extended ECL emission is not necessarily due to the rubrene/oxalate system being presented. Rather, this emission likely occurs because of either the geometry of the droplets themselves or the thickness of the liquid|liquid interface between the aqueous droplet and the organic bulk solution.

To optimize this rubrene/oxalate ECL system, we investigated specific experimental parameters. In a series of experiments, we measured the ECL emission intensity under varied concentrations of rubrene in the organic bulk phase and, separately, different concentrations of oxalate in the aqueous droplet phase. To carry out these experiments, the experimental setup shown in Figures 1a and S1 was used. The rubrene concentration was varied from 2 to 10 mM, and the ECL emission intensity was recorded at each of these concentrations. To measure the ECL emission intensity, we selected five regions of interest (ROIs) in the triphasic boundary-confined ECL signal to extract five different values for the ECL emission intensity. These values are then plotted in Figure 2d, where it is shown that the ECL intensity increases as the rubrene concentration increases from 2 to 6 mM. Then, at rubrene concentrations of 6 mM and higher, the ECL intensity plateaus. Separately, identical experiments were performed to investigate the influence of the oxalate concentration on the ECL emission intensity. In these experiments, the oxalate concentration was increased from 20 to 100 mM, with the results presented in Figure 2e. We observed that when the oxalate concentration was between 20 and 60 mM, the ECL intensity remained relatively stable and dim. However, there was a sharp rise in ECL intensity when the oxalate concentration increased from 60 to 100 mM. Considering these results, concentrations of 10 mM rubrene and 100 mM oxalate were used in all further experimentation to maximize the ECL emission and to remain consistent between phases by saturating both phases with their respective ECL reagents. Figures S11 and S12 depict the ROIs selected for the calculation of the ECL intensity for each of the rubrene and oxalate concentrations, respectively.

In a separate study, we investigated the influence of the applied potential on the emission intensity for the ECL system. As shown in Figure 1c, when CV is carried out to drive the ECL emission, the ECL intensity is maximized when the applied potential is between 1.4 and 1.6 V vs Ag/AgCl/1 M KCl. To investigate if the waveform applied influences at what potential the ECL intensity is maximized, we conducted experiments where potentials ranging from 0.8 to 2.0 V vs Ag/AgCl/1 M KCl were applied for 60 s. The same method of analysis was used as described for the rubrene and oxalate concentration experiments, with the selected ROIs included in Figure S13. As shown in Figure 2f, the ECL intensity increases with the applied potential magnitude until 1.4 V, where it peaks. Then, as the applied potential increases from 1.4 to 2.0 V, the ECL intensity decreases. Thus, it appears that 1.4 V is the optimal potential to apply to maximize the ECL signal, regardless of the shape of the applied waveform. Moreover, by applying potentials of at least 1.4 V, it allows us to avoid the influence of potential drops that may arise from using the large working electrodes (i.e., ITO). Additionally, by applying an overpotential and maintaining 100 mM supporting electrolyte in both the aqueous and organic phases, we minimized any influence of the *iR* drop across the liquid|liquid interface.

To explore if this triphasic boundary-confined ECL emission can extend from single droplets to a microdroplet array, we prepared an emulsion consisting of 5 mL of an aqueous continuous phase of 100 mM sodium oxalate and 100 mM KCl and 100 μL of an organic droplet phase of 10 mM rubrene and 100 mM TBAP in 1,2-DCE. The solution was emulsified via a horn sonicator and then immediately pipetted into an ITO opto-electrochemical cell, as depicted in the first schematic of Figure 3a. After about an hour of waiting, the emulsion settled, so that the ITO was covered with organic microdroplets. This is shown in the second schematic of Figure 3a. Before we performed ECL microscopy, we took a BF micrograph, shown in the frame labeled “BF” in the upper left corner of Figure 3b. To carry out the ECL microscopy, we held a potential of 1.6 V versus Ag/AgCl/1 M KCl for 300 s. Figure S14 shows the amperometric *i*–*t* curve corresponding to Figure 3b. The resulting ECL micrographs captured in this experiment are included in Figure 3b, wherein it can be observed that numerous droplet triphasic boundaries are illuminated. At 0.000 s, or the time at which the potential is initially applied, ECL emission immediately occurs, as seen in the first ECL micrograph of Figure 3b. Then, by 5.360 s, we captured the ECL micrograph with the highest intensity emission and the most illuminated triphasic boundaries. As potential is continuously applied, the ECL intensity of later micrographs continues to decrease, with some illuminated triphasic boundaries disappearing. The ECL emission largely ebbs by the last ECL micrograph at 175.157 s. The advantage of using ECL microscopy on this system is most obvious when comparing the BF micrograph to the ECL micrograph at 5.360 s. In the BF micrograph, limited information about the system can be elucidated, as it is difficult to determine exactly where the droplets are contacting the electrode surface. Conversely, in the ECL micrograph, the triphasic boundaries of many of the settled droplets are illuminated, revealing the outline of the droplets’ contact areas.

**Figure 3:**
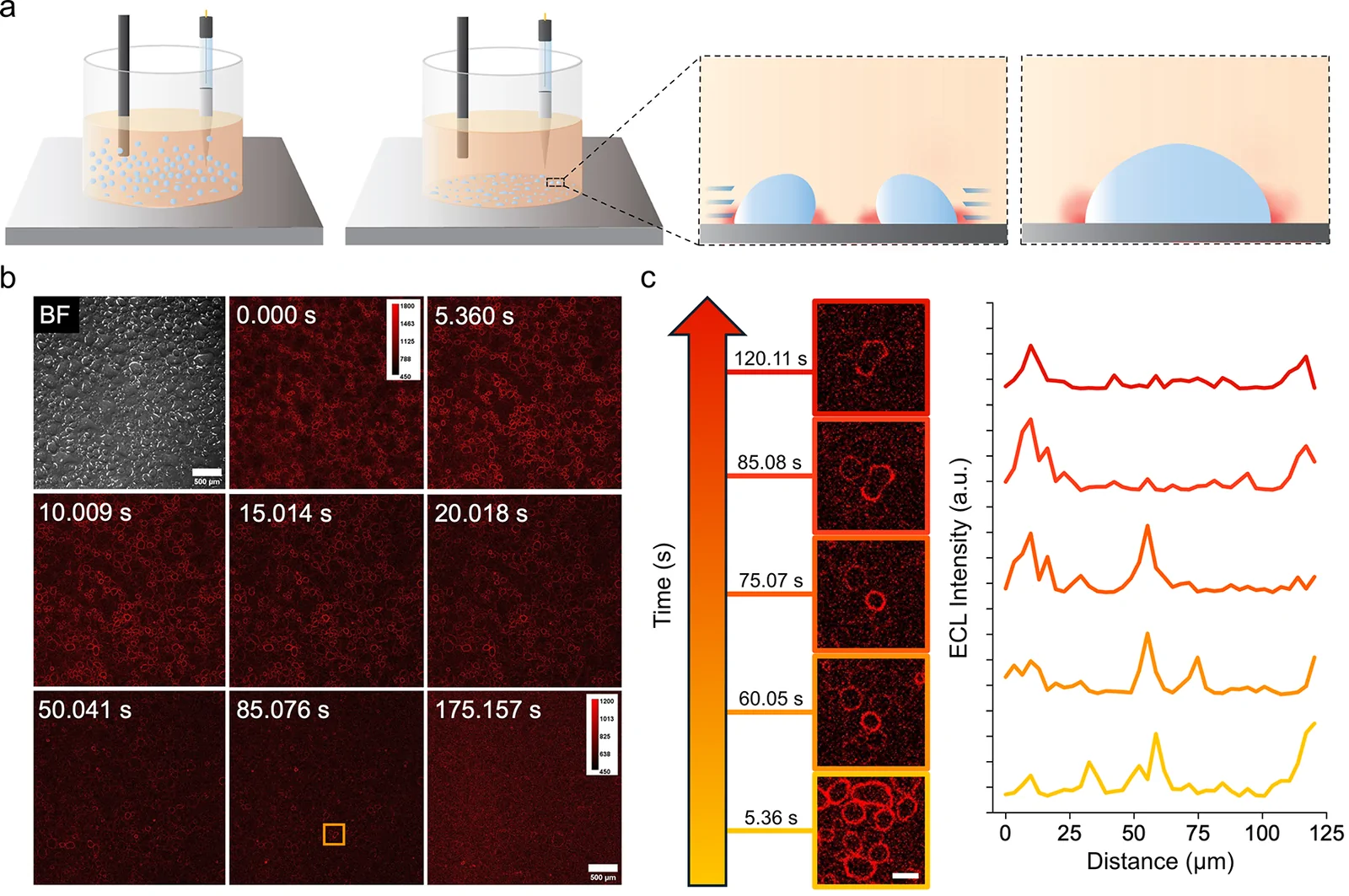
(a) Schematic of the experimental setup, where, first, an emulsion is added into the opto-electrochemical cell (left) and allowed to sit until the emulsion has settled (right). In the inset graphics, a droplet coalescence event is depicted where two separate droplets initially begin moving toward each other (left graphic) until they coalesce into one, larger droplet (right graphic). (b) Series of ECL micrographs illuminating triphasic boundaries within a microdroplet array over time. The emulsion was composed of ca. 100 μL of an organic droplet phase with 10 mM rubrene and 100 mM TBAP within 1,2-DCE and 5 mL of an aqueous, continuous phase with 100 mM sodium oxalate and 100 mM KCl. The emulsion was sonicated with a horn sonicator and then left to settle on an ITO working electrode within an ITO opto-electrochemical cell. To drive the ECL reaction, an amperometric *i*–*t* curve was held for 300 s at 1.6 V vs Ag/AgCl/1 M KCl. The specific experimental details are also included in the Materials and Methods section of the Supporting Information. The scale bars correspond to 500 μm. (c) ECL micrographs and line scans over the course of a droplet coalescence event. The ECL micrographs were inset from the series of micrographs shown in (b), with the orange box in the 85.076 s micrograph indicating where the ECL micrographs in (c) are from. The scale bars correspond to 100 μm.

Within the ECL experiment depicted in Figure 3b, it is demonstrated that this rubrene/oxalate ECL system can selectively light up triphasic boundaries within a microdroplet array. Additionally, this experiment validates that ECL emission is observed regardless of whether the droplet phase is organic and the continuous phase is aqueous, as in Figure 3b, or if the droplet phase is aqueous and the continuous phase is organic, as seen in Figures 1 and 2. Furthermore, this experiment reveals a couple of curious observations. In the ECL micrographs collected at 0.000 and 5.360 s, it appears that the triphasic boundaries at smaller droplets preferentially exhibit ECL emission over the larger droplets. The ECL emission for certain larger (arbitrarily defined as *d* ≥ 250 μm) droplets is not visible until the ECL micrographs collected at 10.009, 15.014, and 175.157 s. Although the emission from the larger droplets remains dim in all the aforementioned ECL micrographs, the outline of a handful of these droplets can be most clearly seen in the 175.157 s ECL micrograph. Another observation is that each of the ECL micrographs is unique in which the triphasic boundaries are visible. For example, when comparing the ECL micrographs collected at 0.000 and 5.360 s, many more triphasic boundaries are illuminated in the latter than in the former. This variance in the ECL emission visible across the ECL micrographs could be caused by differences in the orientation of the droplets on the electrode, specifically, in the droplet contact angles. Differences in the contact angles can influence the mass transfer kinetics of the ECL reagents in both phases, which then affects the intensity of the ECL emission at the droplet|bulk|electrode boundary. Moreover, a separate hypothesis as to why the ECL emission at larger droplets is both much dimmer and delayed as opposed to smaller droplets could be that the confinement effects are less prevalent in larger droplets. Confinement effects have previously been implicated in amplifying ECL signals, typically in microdroplets,^57^ due to the confined environments preventing the diffusion of ECL reagents away from the electrode, causing the local concentration to rise, hence increasing the probability of the luminophore and coreactant molecules meeting and reacting to emit light. Since larger droplets have a larger area for the ECL reagents to diffuse away from the triphasic boundary than smaller droplets, this could be contributing to the described differences in ECL emission.

The effectiveness of this ECL system to interrogate dynamic triphasic boundary movement is further demonstrated in Figure 3c, in which we visualized a droplet coalescence event in real time. This droplet coalescence event occurred within the same microdroplet array ECL trial depicted in Figure 3b. The coalesced droplet is visible in the ECL micrograph taken at 85.076 s in Figure 3b and is highlighted with an orange box surrounding it. To map the droplet coalescence event over time, we took a line scan from one end of the coalesced droplet to the other. The line along which the line scan was taken is shown in Figure S15 and is also represented in the ECL micrograph at 5.36 s in Figure 3c. At 60.05 s, increased intensity ECL signals reveal the triphasic boundaries of the two droplets, with one droplet (lower left) having one side intersecting the line at ca. 10 s and the other intersecting at approximately 55 s. Within this same line scan, the other droplet (upper right) intersects the line at ∼75 and 120 s; however, this droplet is observably much dimmer. At 75.07 s, the ECL signal from the upper right droplet disappears entirely, while the intensity of the lower left droplet increases. By 85.08 s, the droplets have coalesced, resulting in the ECL signal at 75.07 s vanishing but the signal at 120.11 s returning as further evidence for the formation of the new, coalesced droplet.

Thus far, we have demonstrated that this rubrene/oxalate ECL system can spatially map and track triphasic (droplet, bulk, and electrode) boundary locations in real time. However, the triphasic boundaries interrogated in this study have been limited to single microdroplets and microdroplet arrays. To explore if this technique can be extended to bulk, multiphase systems, an experiment was performed at a bulk liquid|liquid|electrode interface. In this experiment, which is shown in Figure 4a, a glassy carbon rod (*d* = 2 mm) working electrode (center electrode) was placed in the center of a glass opto-electrochemical cell so that it pierced through a bulk liquid|liquid interface. This interface was created by first pipetting in approximately 2 mL of the organic phase (represented as orange in Figure 4a) consisting of 10 mM rubrene and 100 mM TBAP in 1,2-DCE to the cell, followed by the addition of about 2 mL of the aqueous phase (shown as blue in Figure 4a), composed of 100 mM oxalate and 100 mM KCl. To complete the electrochemical cell, a GC rod counter electrode (left electrode) was placed in the cell and an Ag/AgCl/1 M KCl reference electrode was connected to the system via an agarose salt bridge (right electrode).

**Figure 4:**
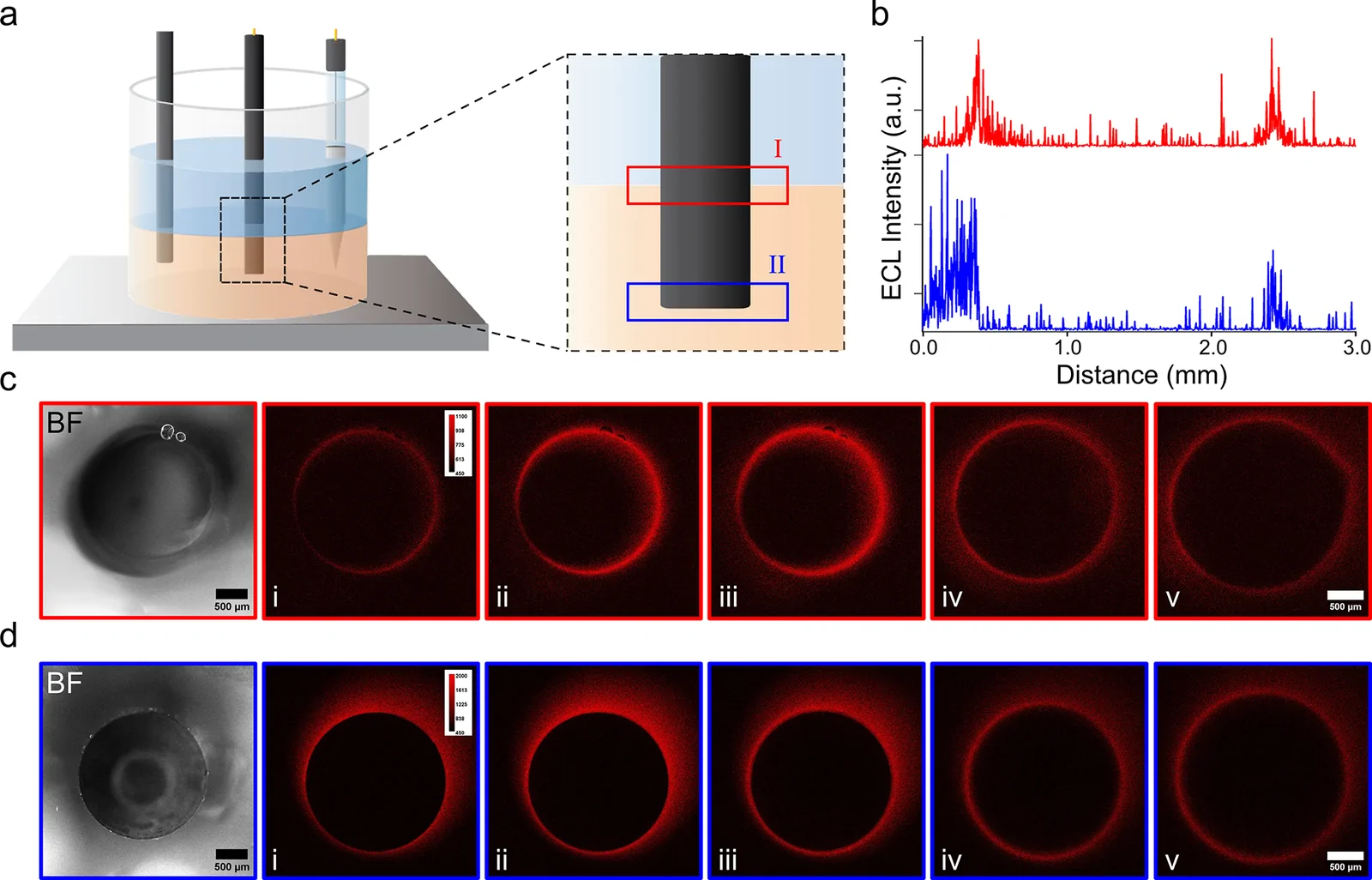
(a) Schematic of the experimental setup where a bulk liquid|liquid|electrode triphasic boundary was interrogated. The system was composed of a bottom organic phase with 10 mM rubrene and 100 mM TBAP in 1,2-DCE and a top aqueous phase with 100 mM sodium oxalate and 100 mM KCl. Approximately 2 mL of each phase was added to the glass opto-electrochemical cell, and a GC rod working electrode was placed into the system so that it penetrated both phases, with the end of the rod immersed in the bulk, organic phase. The inset graphic highlights two imaging planes on the GC rod electrode where ECL microscopy was carried out. Region I (red) is at the triphasic boundary, whereas region II (blue) is at the end of the GC rod that is immersed in the bulk organic phase. (b) Line scans of the two different regions, taken from the ECL micrographs shown in Figure 4c­(ii) (red) and Figure 4d­(ii) (blue). (c) BF micrograph and the corresponding ECL micrographs with the focus at Region I. To drive the ECL reaction, we carried out CV in the potential window of 0 to 1.8 V vs Ag/AgCl/1 M KCl at a scan rate of 50 mV/s. We took ECL micrographs at the following potentials: (i) 1.05, (ii) 1.10, (iii) 1.15, (iv) 1.45, and (v) 1.75 V on the backsweep of the CV. (d) Both a BF micrograph and a series of ECL micrographs with the focus at Region II. We performed CV in the potential window of 0 to 1.8 V at the scan rate of 50 mV/s. We captured ECL micrographs at the following potentials: (i) 1.17, (ii) 1.22, (iii) 1.27, (iv) 1.52, and (v) 1.72 V.

To visualize the triphasic boundary between the bulk aqueous phase, the bulk organic phase, and the GC rod working electrode, we selected two imaging planes. The first was at the triphasic boundary itself, represented in Figure 4a as Region I (red), and the second was at the bottom of the glassy carbon rod electrode, which was immersed in the organic phase, represented as Region II (blue). We selected these imaging planes to investigate whether we could elucidate differences in the ECL emission layer morphologies between them. A series of ECL micrographs that we focused on Region I is shown in Figure 4c, while Figure 4d shows a series of ECL micrographs that we focused on Region II. Strikingly, clear differences in the appearance of the emission layer are visible between these imaging planes when comparing the first three micrographs (i, ii, iii) of Figure 4c,4d. When we optically focused on the triphasic boundary, as in the case of Figure 4c, we observed a “ring” of ECL emission immediately surrounding the electrode. This is expected, as the ECL reaction primarily is surface-confined, wherein the photons are emitted in the area immediately surrounding the electrode. It is important to highlight that due to the optical setup orientation, wherein the electrode is being imaged from below, diffraction of photons causes some of the ECL emission layer to appear both inward and outward from the triphasic boundary when focused on Region I. Additionally, the GC rod working electrode, although oriented as vertically as possible, appears to have been slightly askew, resulting in the expected 2D axisymmetric ECL emission being nonuniform.

Conversely, when optically focused on the end of the GC rod, millimeters away from the triphasic boundary, as in the case of Region II, the ECL micrographs demonstrate a very different emission layer morphology. As shown in the first three ECL micrographs of Figure 4d, the ECL emission layer appears to have a sharp emission immediately surrounding where the electrode is, which then quickly fades in the regions farther away from the electrode surface. We expected this ECL emission layer morphology because when focused at Region II in this optical setup, the end of the electrode itself blocks the ECL emission coming from the triphasic boundary. This is akin to what is observed in a solar eclipse, wherein the moon blocks the light coming from the sun, so the sun instead appears as a pronounced ring around the moon, surrounded by a larger diffuse area of light. Hence, as seen in Figure 4d­(ii), we observed no ECL emission in the area where the electrode is, as the electrode blocks the light from reaching the detector, while in Figure 4c­(ii), diffuse light is observed. This observation is further supported and made apparent by Figure 4b, which shows the results of line scans taken through Figure 4c­(ii) (top plot, red) and Figure 4d­(ii) (bottom plot, blue). The CVs corresponding to Figure 4 are included in Figure S16, and the location of the lines used for the line scans is shown in Figure S17. As exhibited in Figure 4b, the ECL intensity vs distance plot, the ECL emission at the triphasic boundary in Figure 4c­(ii) (top plot, red) appears as two emission signals that both exhibit a central, maximum ECL intensity with a lower intensity, tapering emission on either side. The first emission signal is centered around ca. 0.3 mm, and the second signal peaks at around 2.3 mm. The distance between these signals matches the measured diameter of the electrode, verifying that this signal originates at the electrode. Furthermore, it is critical to emphasize the shape of these ECL signals, where the elevated yet tapering emission on either side of the max ECL intensity can be attributed to the diffracted photons on either side of the triphasic boundary that create the diffuse ECL emission region that is visible in Figure 4c­(ii). In comparison, the line scan of Figure 4d­(ii) (bottom plot, blue) within Figure 4b depicts a much different shape for the ECL emission layer. Notably, for both observed signals within Figure 4d­(ii) that are shown in Figure 4b, there is a maximum ECL intensity in which one side, the side oriented outside of the electrode, has a gradual decrease in the signal, whereas the side toward the inside of the electrode has an immediate, steep drop-off in the signal. This steep drop-off in the ECL signal supports the hypothesis that the end of the electrode blocks the light illuminated at the triphasic boundary.

Interestingly, when comparing the ECL micrographs to the BF micrographs in both Figure 4c,4d, the power of imaging this system with triphasic boundary-confined ECL microscopy becomes apparent. To clarify, in the BF micrograph of Figure 4c, the micrograph appears to be slightly warped, and it is not obvious where the triphasic boundary is located. The advent of two bubbles being settled on the surface of the electrode aids in the search to locate the boundary; however, the bubbles themselves are also a bit distorted due to their reflection off the electrode. However, as seen in Figure 4c­(i),(ii), by selectively emitting photons at the triphasic boundary via this ECL reaction, the boundary can be resolved, overcoming the optical artifacts observed in the BF that resulted in the distortion. Similarly, in Figure 4d, the ECL emission morphology reveals that the glassy carbon rod working electrode was not exactly vertical, which would not have been known by merely looking at the BF micrograph. Moreover, by being able to elucidate differences in the ECL emission layer between the different imaging planes along the glassy carbon rod working electrode, in the *z*-direction, the use of this rubrene/oxalate ECL imaging platform extends from the 2D space to the 3D space.

## Conclusions

In this study, we carefully designed an ECL system using rubrene as a luminophore and oxalate as a coreactant that limits ECL emission to triphasic boundaries. Specifically, by taking advantage of the differences in the solubility of rubrene and oxalate, the interface between the electrode, the organic phase, and the aqueous phase is the only place in the system where the ECL reagents could be oxidized and react with each other, enabling this triphasic boundary-confined ECL emission. Here, to demonstrate the use of this ECL imaging platform, we interrogated the triphasic boundary of single microdroplets (Figures 1 and 2), a microdroplet array (Figure 3), and a bulk liquid|liquid|electrode interface (Figure 4). Using this rubrene/oxalate ECL system, we illuminated triphasic boundaries within complex, multiphase systems, revealing their spatial location within the system (Figure 3). Moreover, we tracked and visualized the dynamic movement of microdroplets, specifically, both a reorganization event (Figure 2) and a coalescence event (Figure 3), in real time. Even more strikingly, we elucidated differences between the ECL emission layer morphology at different *z*-direction imaging planes using this rubrene/oxalate system, demonstrating that this system transcends the 2D restriction that many ECL imaging systems have by being able to highlight differences in the triphasic boundary orientation within a 3D space.

As multiphase systems are at the forefront of many curious chemistry investigations and industrial processes, new methods are necessary to develop a more complete physicochemical framework for these elusive, complex environments. Additionally, to be able to better understand dynamic processes within these multiphase, microconfined systems, especially on an electrified interface, such as droplet reorganization events and phase- and charge-transfer processes, innovative studies need to be designed that isolate their interrogation to the triphasic boundaries wherein these processes occur. In this study, we present an ECL imaging technique that selectively illuminates triphasic boundaries, demonstrating that this technique can image these boundaries in both bulk and microconfined multiphase systems. Moreover, we show that this technique can also map and track the dynamic movement of boundaries in real time, revealing the physicochemical properties of multiphase systems. Our framework presents a unique and promising platform to interrogate multiphase systems, which may lay the foundation for future innovative imaging methodologies to illuminate new and exciting chemistries.

## Supplementary Material















## Data Availability

The data that support the findings of this study are available from the corresponding authors upon request.

## Notes

Materials and methods, selected regions of interest for the data analysis, cyclic voltammetry, lines used for the line scans, and further control experiments (PDF)
Corresponds to the main text Figure 1b. The droplet has 100 mM sodium oxalate and 100 mM KCl in H_2_O. The bulk phase contains 6 mM rubrene and 100 mM TBAP within 1,2-DCE. The scale bar is 500 μm (MOV)
Corresponds to the main text Figure 1c. The droplet has 100 mM sodium oxalate and 100 mM KCl in H_2_O. The bulk phase contains 10 mM rubrene and 100 mM TBAP within 1,2-DCE. The scale bar is 500 μm (MOV)
Corresponds to the main text Figure 2b,c. The droplet has 80 mM sodium oxalate and 100 mM KCl in H_2_O. The bulk phase contains 10 mM rubrene and 100 mM TBAP within 1,2-DCE. The scale bar is 500 μm (MOV)
Corresponds to the main text Figure 3b,c. The droplet phase 10 mM rubrene and 100 mM TBAP in 1,2-DCE. The continuous (or bulk) phase, the aqueous phase, consisted of 100 mM sodium oxalate and 100 mM KCl in H_2_O. The scale bar is 500 μm (MOV)
Corresponds to the main text Figure 4c. ECL microscopy of a bulk liquid|bulk liquid|electrode triphasic boundary with the focus of the camera on the triphasic boundary. The bottom, organic phase was composed of 10 mM rubrene and 100 mM TBAP in 1,2-DCE, while the top, aqueous phase consisted of 100 mM sodium oxalate and 100 mM KCl. The scale bar is 500 μm (MOV)
Corresponds to the main text Figure 4 d. ECL microscopy of a bulk liquid|bulk liquid|electrode triphasic boundary with the focus of the camera at the end of the GC rod electrode. The bottom, organic phase was composed of 10 mM rubrene and 100 mM TBAP in 1,2-DCE, while the top, aqueous phase consisted of 100 mM sodium oxalate and 100 mM KCl. The scale bar is 500 μm (MOV)
