# Supplementary material for: Rubrene–Oxalate Electrochemiluminescence Reveals Physicochemical Properties of Triphasic Boundaries
Source: Chem Biomed Imaging. 2026 Apr 3;4(9):2041–53. doi: 10.1021/cbmi.5c00295 (PMC13625697; doi:10.1021/cbmi.5c00295)
Supplement: Supplementary file 1 [file im5c00295_si_001.pdf]

Supporting Information for:

Rubrene-Oxalate Electrochemiluminescence Reveals Physicochemical Properties of Triphasic Boundaries

Megan L. Hill[a§], Mackenzie P. Stockwell[a§], Jeffrey E. Dick[a*]

[a]Department of Chemistry, Purdue University, West Lafayette, IN, 47907, United States
*To whom correspondence should be addressed: jdick@purdue.edu
[§]Indicates Equal Contribution

Description	Page(s)
**Materials and Methods**	S2 – S6
**Figure S1.** Brightfield and ECL Microscopy Experimental Set-Up.	S7
**Figure S2.** Correlated Cyclic Voltammogram for Figure 1B.	S8
**Figure S3.** Proposed Mechanism of Oxalate Phase Transfer.	S9
**Figure S4.** ECL Microscopy with Different Organic Phase Supporting Electrolytes.	S10
**Figure S5.** Cyclic Voltammetry of Rubrene and Oxalate Bulk Solutions.	S11
**Figure S6.** Control: Cyclic Voltammetry and ECL Microscopy without Rubrene.	S12
**Figure S7.** Control: Cyclic Voltammetry and ECL Microscopy without Oxalate.	S13
**Figure S8.** BF and ECL Microscopy of Different-Sized Droplets.	S14
**Figure S9.** Correlated Cyclic Voltammogram for Figure 2B and Figure 2C.	S15
**Figure S10.** Line used for the Line Scans in Figure 2C.	S16
**Figure S11.** Selected ROIs for ECL Intensity Measurements at Different Rubrene Concentrations.	S17
**Figure S12.** Selected ROIs for ECL Intensity Measurements at Different Oxalate Concentrations.	S18
**Figure S13.** Selected ROIs for ECL Intensity Measurements at Different Applied Potentials.	S19
**Figure S14.** Amperometric i-t curve corresponding to Figure 3.	S20
**Figure S15.** Line used for Figure 3C line scans.	S21
**Figure S16.** Cyclic Voltammogram corresponding to Figure 4.	S22
**Figure S17.** Lines used for the Line Scans in Figure 4B.	S23
**SI Movie Descriptions**	S24

Materials and Methods

Materials, Reagents, and Solution Preparation

Rubrene (≥ 98%) and Sodium Oxalate (≥ 99.5%) were purchased from Sigma Aldrich. Moreover, organic salts of tetra-n-butylammonium hexafluorophosphate (99%), tetra-n-butylammonium bromide (97%), and tetra-n-butylammonium chloride (99%) were also purchased from Sigma Aldrich. Potassium chloride (KCl, certified ACS), tetra-n-butylammonium perchlorate (99+%), 1,2-dichloroethane (1,2-DCE, HPLC grade, >99%), and tris(2,2'-bipyridyl)ruthenium(II) chloride hexahydrate ($[Ru(bpy)_3][Cl_2]\cdot 6H_2O$, 98%) were purchased from ThermoFisher Scientific. The tris(2,2'-bipyridyl)ruthenium(II) chloride hexahydrate ($[Ru(bpy)_3][Cl_2]\cdot 6H_2O$, 98%) was stored in a dark container under nitrogen in a desiccator. All chemicals were used *as received* without any further purification steps, and all solutions were prepared with ultrapure water (Millipore Milli-Q, 18.2 MΩ cm).

Opto-electrochemical Cell Fabrication

In this study, two types of opto-electrochemical cells were fabricated: glass cells and indium tin-doped oxide (ITO) cells. Primarily, ITO opto-electrochemical cells were used, as they were necessary for all single microdroplet and microdroplet array trials). The ITO opto-electrochemical cells were fabricated using an ITO working electrode (Qunguan Electronics, China, 100 mm x 100 mm x 1.1 mm) and a glass cylinder (Scientific Glass Blowing Lab, Purdue University Department of Chemistry; internal diameter (ID) = 19 mm and height (H) = 20 mm). To fabricate the ITO opto-electrochemical cells, a glass cylinder is epoxied onto a square laser-cut *ca.* 25 mm by 25 mm piece of ITO. Gorilla Epoxy (Gorillatough.com, USA) is used for this purpose. The ITO opto-electrochemical cells are then dried and cured overnight, with the epoxy seal between the ITO working electrode and the cylinder checked by filling the cell with ultrapure water (Millipore Milli-Q, 18.2 MΩ cm) prior to use in experimentation. To electrically connect the ITO working electrode to the potentiostat, a piece of copper wire is placed on the ITO to which the potentiostat lead is connected.

Alternatively, glass opto-electrochemical cells were also fabricated for use with the glassy carbon (GC) rod working electrode experiments. The glass cells were fabricated similarly to the ITO opto-electrochemical cells described above wherein a glass cylinder (Scientific Glass Blowing Lab, Purdue University Department of Chemistry; ID = 19 mm and H = 20 mm) is epoxied using Gorilla Epoxy (Gorillatough.com, USA) onto a thin glass coverslip (VWR, Germany; 24 mm x 40 mm x 130 – 160 µm). After epoxying, the cells are left to dry and cure overnight. Before use in experiments, the epoxy seal between the coverslip and the cylinder is checked by filling the cell with ultrapure water (Millipore Milli-Q, 18.2 MΩ cm).

Electrochemistry and Optical Microscopy

To perform the electrochemistry required to drive the electrochemiluminescence (ECL) reactions, a model 601E CHI potentiostat (CH Instruments, Austin, Texas) was used. For all experiments, a GC rod (d = 2 mm and L = 10 cm, Metrohm USA, Riverview, FL) was used as the counter electrode. Moreover,

for all experiments, an Ag/AgCl/1 M reference electrode was used that was connected to the electrochemical cell using an agarose salt bridge. The agarose salt bridge was fabricated by filling a glass pipette with agarose, leaving space in the top for the reference electrode to sit. This space was filled with 1 M KCl, in which the reference electrode is submerged. For the experiments included in the main text figures of Figure 1, Figure 2, and Figure 3, an ITO (procured from Qunguan Electronics, China) working electrode was used. Whereas, for main text Figure 4, a GC rod *(d* = 2 mm and *L* = 10 cm, Metrohm USA, Riverview, FL) was used as the working electrode.

To carry out the optical microscopy necessary for ECL microscopy, a Nikon Eclipse Ti2 inverted optical microscope (Nikon, Japan) and an EMCCD iXon 888 camera (Andor Technology Ltd., Belfast, UK) were used. Moreover, brightfield imaging was performed using a Complementary Metal-Oxide Semiconductor (CMOS) camera from Hamamatsu (Orca-Quest qC-MOS C15550-20UP, Hamamatsu, Japan) with an external MKII (Nikon, Japan) light source. For the ECL microscopy, the EMCCD camera was used with the following parameters unless otherwise specified: 1.0 s exposure time, 10 MHz quality mode, 400 electron multiplier (EM) gain, 4.22 μs shift speed, and a temperature that varied between -75°C and -85ºC. Additionally, unless otherwise stated, each experiment used a 4x objective with a working distance of 20 mm and a numerical aperture (NA) of 0.20. The *Specific Experimental Details* section below further specifies the experimental setup and parameters used for each of the main text experiments.

Software and Data Analysis

All data analysis was performed in either Matlab, OriginPro, NIS Elements, Fiji or ImageJ. To acquire all images and videos, the NIS Elements Software (Nikon, Japan) was used whereas all capture videos and images were analyzed in Fiji or ImageJ (National Institutes of Health Freeware, Bethesda Maryland USA). OriginPro (OriginLab Corporation, Northampton, MA) or Matlab (MathWorks, Natick, Massachusetts USA) were used to make all graphs and plots. The *Specific Experimental Details* section below further specifies the analysis performed for each experiment.

Specific Experimental Details

Figure 1B: In this experiment, ECL microscopy of a *ca.* 2 μL aqueous droplet composed of 100 mM sodium oxalate and 100 mM KCl on an ITO working electrode was performed. The droplet was immersed in a bulk phase of 6 mM rubrene and 100 mM TBAP within 1,2-DCE, all within an ITO opto-electrochemical cell. The counter electrode was a GC rod and the reference electrode was an Ag/AgCl/1 M KCl electrode connected to the system via an agarose salt bridge. To drive the ECL reaction, cyclic voltammetry was carried out, sweeping from 0 to 1.8 V vs Ag/AgCl/1 M KCl at a scan rate of 50 mV/s. For the ECL microscopy, an EMCCD camera with a 4x objective and a 1x zoom was used with the following parameters: Gain 2, 10 MHz readout speed, 1 second exposure, and an EM gain of 400.

Figure 1C: In this experiment, ECL microscopy of a *ca.* 2 μL aqueous droplet composed of 100 mM sodium oxalate and 100 mM KCl on an ITO working electrode was performed. The droplet was immersed in a bulk phase of 10 mM rubrene and 100 mM TBAP within 1,2-DCE, all within an ITO opto-electrochemical cell. The counter electrode was a GC rod, and the reference electrode was an Ag/AgCl/1 M KCl electrode connected to the system via an agarose salt bridge. To drive the ECL reaction, cyclic voltammetry was carried out, sweeping from 0 to 1.8 V vs Ag/AgCl/1 M KCl at a scan rate of 50 mV/s.

For the ECL microscopy, an EMCCD camera with a 4x objective and a 1x zoom was used with the following parameters: Gain 2, 10 MHz readout speed, 1 second exposure, and an EM gain of 400.

To determine the average intensity of ECL emission for the correlated ECL intensity and cyclic voltammetry plot, a region of interest (ROI) was drawn using a ring ROI in the NIS Elements Software (Nikon, Japan). Specifically, a ring ROI was drawn around the edge of the droplet where the triphasic boundary was located. Once the ring ROI was drawn, a time measurement of the average ECL intensity within the ROI was carried out and then subsequently background-subtracted. To perform the background subtraction, first, the mean average intensity across the ROI for ~15 frames before the application of potential was measured. Then, the value of this background, the average mean ECL intensity, was subtracted from all the frames within the captured video of the trial, setting the background to a value of 0. Furthermore, to determine the limit of detection (LOD) for the correlated ECL intensity and cyclic voltammetry plot, the standard deviation of the same selected frames used in the background subtraction was calculated. The LOD was then set to 3 times this standard deviation.

Figure 1D: In this experiment, ECL microscopy of a *ca.* 2 μL aqueous droplet composed of 100 mM sodium oxalate and 100 mM KCl on an ITO working electrode was performed. The droplet was immersed in a bulk phase of 10 mM rubrene and 100 mM TBAP within 1,2-DCE, all within an ITO opto-electrochemical cell. The counter electrode was a GC rod, and the reference electrode was an Ag/AgCl/1 M KCl electrode connected to the system via an agarose salt bridge. To drive the ECL reaction, cyclic voltammetry was carried out, sweeping from 0 to 1.8 V vs Ag/AgCl/1 M KCl at a scan rate of 50 mV/s. To capture the brightfield (BF) image, a CMOS camera was used with an external MKII light source illuminating the droplet. For the ECL microscopy, an EMCCD camera with a 4x objective and a 1x zoom was used with the following parameters: Gain 2, 10 MHz readout speed, 1 second exposure, and an EM gain of 400. To make the overlay micrograph, Fiji was used with the ECL micrograph placed over the BF micrograph at ~ 60% opacity.

Figure 2B & Figure 2C: In this experiment, ECL microscopy of a *ca.* 2 μL aqueous droplet composed of 80 mM sodium oxalate and 100 mM KCl on an ITO working electrode was performed. The droplet was immersed in a bulk phase of 10 mM rubrene and 100 mM TBAP within 1,2-DCE, all within an ITO opto-electrochemical cell. The counter electrode was a GC rod, and the reference electrode was an Ag/AgCl/1 M KCl electrode connected to the system via an agarose salt bridge. To drive the ECL reaction, cyclic voltammetry was carried out, sweeping from 0 to 1.8 V vs Ag/AgCl/1 M KCl at a scan rate of 50 mV/s. For the ECL microscopy, an EMCCD camera with a 4x objective and a 1x zoom was used with the following parameters: Gain 2, 10 MHz readout speed, 1 second exposure, and an EM gain of 400. For the line scan in Figure 2C, the line was taken across the droplet, as shown in **Figure S10**.

Figure 2D: In this series of experiments, ECL microscopy of a *ca.* 2 μL aqueous droplet composed of 100 mM sodium oxalate and 100 mM KCl on an ITO working electrode was performed. The droplet was immersed in a bulk phase of rubrene at varying concentrations and 100 mM TBAP within 1,2-DCE, all within an ITO opto-electrochemical cell. The counter electrode was a GC rod and the reference electrode was an Ag/AgCl/1 M KCl electrode connected to the system via an agarose salt bridge. The concentrations of rubrene interrogated were: 2 mM, 4 mM, 6 mM, 8 mM, and 10 mM. To carry out the ECL reaction, cyclic voltammetry was carried out, sweeping from 0 to 1.8 V vs Ag/AgCl/1 M KCl at a scan rate of 50 mV/s. For the ECL microscopy, an EMCCD camera with a 4x objective and a 1x zoom was used with the following parameters: Gain 2, 10 MHz readout speed, 1 second exposure, and an EM gain of 400.

To estimate the average intensity of ECL emission for the ECL intensity vs rubrene concentration plot, five ROIs were selected along the triphasic boundary in each of the trials within the NIS Elements Software (Nikon, Japan). These selected ROIs are shown in **Figure S11**. For each different ROI, a time measurement of the average ECL intensity within the ROI was carried out and then subsequently background-subtracted. To perform the background subtraction, first, the mean average intensity across a square ROI away from the triphasic boundary for ~15 frames before the application of potential was measured. Then, the value of this background, the average mean ECL intensity, was subtracted from all the frames within the captured video of the trial, setting the background to a value of 0. Once the background-subtracted mean ECL intensity was determined for each of the five ROIs, the ROIs were plotted on the ECL intensity vs rubrene concentration graph. Moreover, the limit of detection (LOD) for each of the ROIs was determined by calculating the standard deviation of the same selected frames used in the background subtraction and multiplying the value by 3. The LOD shown on the plot was the LOD with the highest value (or the LOD with the lowest sensitivity) out of each of the ROIs.

<u>Figure 2E:</u> In this series of experiments, ECL microscopy of a *ca.* 2 μL aqueous droplet composed of sodium oxalate at different concentrations and 100 mM KCl on an ITO working electrode was performed. The droplet was immersed in a bulk phase of 10 mM rubrene and 100 mM TBAP within 1,2-DCE, all within an ITO opto-electrochemical cell. The counter electrode was a GC rod, and the reference electrode was an Ag/AgCl/1 M KCl electrode connected to the system via an agarose salt bridge. The concentrations of oxalate tested were: 20 mM, 40 mM, 60 mM, 80 mM, and 100 mM. To carry out the ECL reaction, cyclic voltammetry was performed, sweeping from 0 to 1.8 V vs Ag/AgCl/1 M KCl at a scan rate of 50 mV/s. For the ECL microscopy, an EMCCD camera with a 4x objective and a 1x zoom was used with the following parameters: Gain 2, 10 MHz readout speed, 1 second exposure, and an EM gain of 400.

To estimate the average intensity of ECL emission for the ECL intensity vs oxalate concentration plot, five ROIs were selected along the triphasic boundary in each of the trials within the NIS Elements Software (Nikon, Japan). These selected ROIs are shown in **Figure S12**. For each different ROI, a time measurement of the average ECL intensity within the ROI was carried out and then subsequently background-subtracted. To perform the background subtraction, first, the mean average intensity across a square ROI away from the triphasic boundary for ~15 frames before the application of potential was measured. Then, the value of this background, the average mean ECL intensity, was subtracted from all the frames within the captured video of the trial, setting the background to a value of 0. Once the background-subtracted mean ECL intensity was determined for each of the five ROIs, the ROIs were plotted on the ECL intensity vs rubrene concentration graph. Moreover, the limit of detection (LOD) for each of the ROIs was determined by calculating the standard deviation of the same selected frames used in the background subtraction and multiplying the value by 3. The LOD shown on the plot was the LOD with the highest value (or the LOD with the lowest sensitivity) out of each of the ROIs.

<u>Figure 2F:</u> In this series of experiments, ECL microscopy of a *ca.* 2 μL aqueous droplet composed of 100 mM sodium oxalate and 100 mM KCl on an ITO working electrode at different applied potentials was performed. The droplet was immersed in a bulk phase of 10 mM rubrene and 100 mM TBAP within 1,2-DCE, all within an ITO opto-electrochemical cell. The counter electrode was a GC rod, and the reference electrode was an Ag/AgCl/1 M KCl electrode connected to the system via an agarose salt bridge. To drive the ECL reaction, amperometric i-t curves held for 60 s at different applied potentials were used. The applied potentials were: 0.8 V, 1.0 V, 1.2 V, 1.4 V, 1.6 V, 1.8 V, and 2.0 V vs Ag/AgCl/1 M KCl. For the

ECL microscopy, an EMCCD camera with a 4x objective and a 1x zoom was used with the following parameters: Gain 2, 10 MHz readout speed, 1 second exposure, and an EM gain of 400.

To estimate the average intensity of ECL emission for the ECL intensity vs applied potential plot, five ROIs were selected along the triphasic boundary in each of the trials within the NIS Elements Software (Nikon, Japan). These selected ROIs are shown in **Figure S13.** For each different ROI, a time measurement of the average ECL intensity within the ROI was carried out and then subsequently background-subtracted. To perform the background subtraction, first, the mean average intensity across a square ROI away from the triphasic boundary for ~15 frames before the application of potential was measured. Then, the value of this background, the average mean ECL intensity, was subtracted from all the frames within the captured video of the trial, setting the background to a value of 0. Once the background-subtracted mean ECL intensity was determined for each of the five ROIs, the ROIs were plotted on the ECL intensity vs rubrene concentration graph. Moreover, the limit of detection (LOD) for each of the ROIs was determined by calculating the standard deviation of the same selected frames used in the background subtraction and multiplying the value by 3. The LOD shown on the plot was the LOD with the highest value (or the LOD with the lowest sensitivity) out of each of the ROIs.

Figure 3B & Figure 3C: In this experiment, ECL microscopy of an emulsion composed of *ca.* 100 µL of an organic, droplet phase composed of 10 mM rubrene and 100 mM TBAP within 1,2-DCE and 5 mL of an aqueous, continuous phase consisting of 100 mM sodium oxalate and 100 mM KCl was interrogated. The emulsion was sonicated with a horn-sonicator and then left to settle on an ITO working electrode within an ITO opto-electrochemical cell. The counter electrode was a GC rod and the reference electrode was an Ag/AgCl/1 M KCl electrode connected to the system via an agarose salt bridge. To drive the ECL reaction, an amperometric i-t curve held for 300 s at 1.6 V vs Ag/AgCl/1 M KCl was carried out. To capture the brightfield (BF) image, a CMOS camera was used with an external MKII light source illuminating the settled emulsion. For the ECL microscopy, an EMCCD camera with a 4x objective and a 1x zoom was used with the following parameters: Gain 2, 10 MHz readout speed, 5-second exposure, and an EM gain of 999. For the line scan in Figure 3C, the line was taken across the coalesced droplet, as shown in **Figure S15**.

Figure 4B, Figure 4C, & Figure 4D: In this experiment, ECL microscopy of a bulk liquid|bulk liquid|electrode system was carried out, with Figure 4C focused at the triphasic boundary, while Figure 4D is focused on the end of the working electrode. The bottom, organic phase was composed of 10 mM rubrene and 100 mM TBAP in 1,2-DCE, while the top, aqueous phase consisted of 100 mM sodium oxalate and 100 mM KCl. Approximately 2 mL of each phase was added to the glass opto-electrochemical cell, and a GC rod working electrode was placed into the system so that it penetrated both phases, with the end of the rod immersed in the bulk, organic phase. To drive the ECL reaction, cyclic voltammetry was performed, sweeping from 0 to 1.8 V vs Ag/AgCl/1 M KCl at a scan rate of 50 mV/s. To capture the brightfield (BF) image, a CMOS camera was used with an external MKII light source. For the ECL microscopy, an EMCCD camera with a 4x objective and a 1x zoom was used with the following parameters: Gain 2, 10 MHz readout speed, 1-second exposure, and an EM gain of 400. For the line scans in Figure 4B, the line was taken across the triphasic boundary (approximately the diameter of the GC rod in both cases), as shown in **Figure S17**.

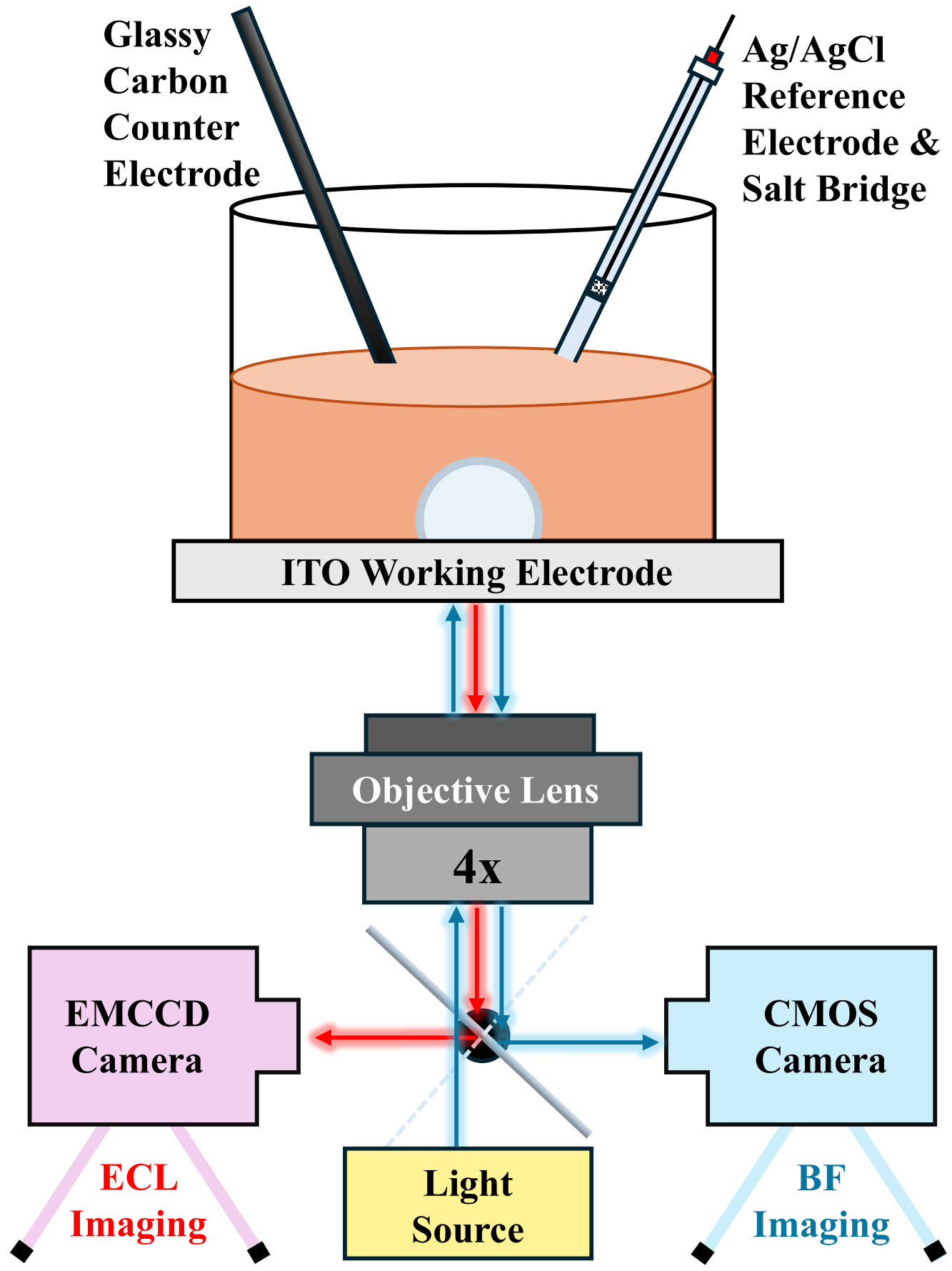

Figure S1. Brightfield and ECL Microscopy Experimental Set-Up. In this experimental set-up, used for the experiments included in Figures 1, 2, and 3, an ITO opto-electrochemical cell was used wherein the optically transparent, conductive ITO was the working electrode. The counter electrode was a GC rod and the reference electrode was an Ag/AgCl/1 M KCl electrode connected to the system via an agarose salt bridge. For BF and ECL imaging, a 4x objective lens with a 1x zoom was employed and focused at the triphasic boundary between the droplet|bulk|ITO electrode. An EMCCD camera was used to acquire ECL micrographs, while a CMOS camera with an external MKII light source was used to capture the BF micrographs. The system shown is an aqueous droplet composed of 100 mM oxalate and 100 mM KCl within a bulk, organic phase of 10 mM rubrene and 100 mM TBAP in 1,2-DCE. See the *Specific Experimental Details* section above for a more detailed description of each of the experiments included in the main text.

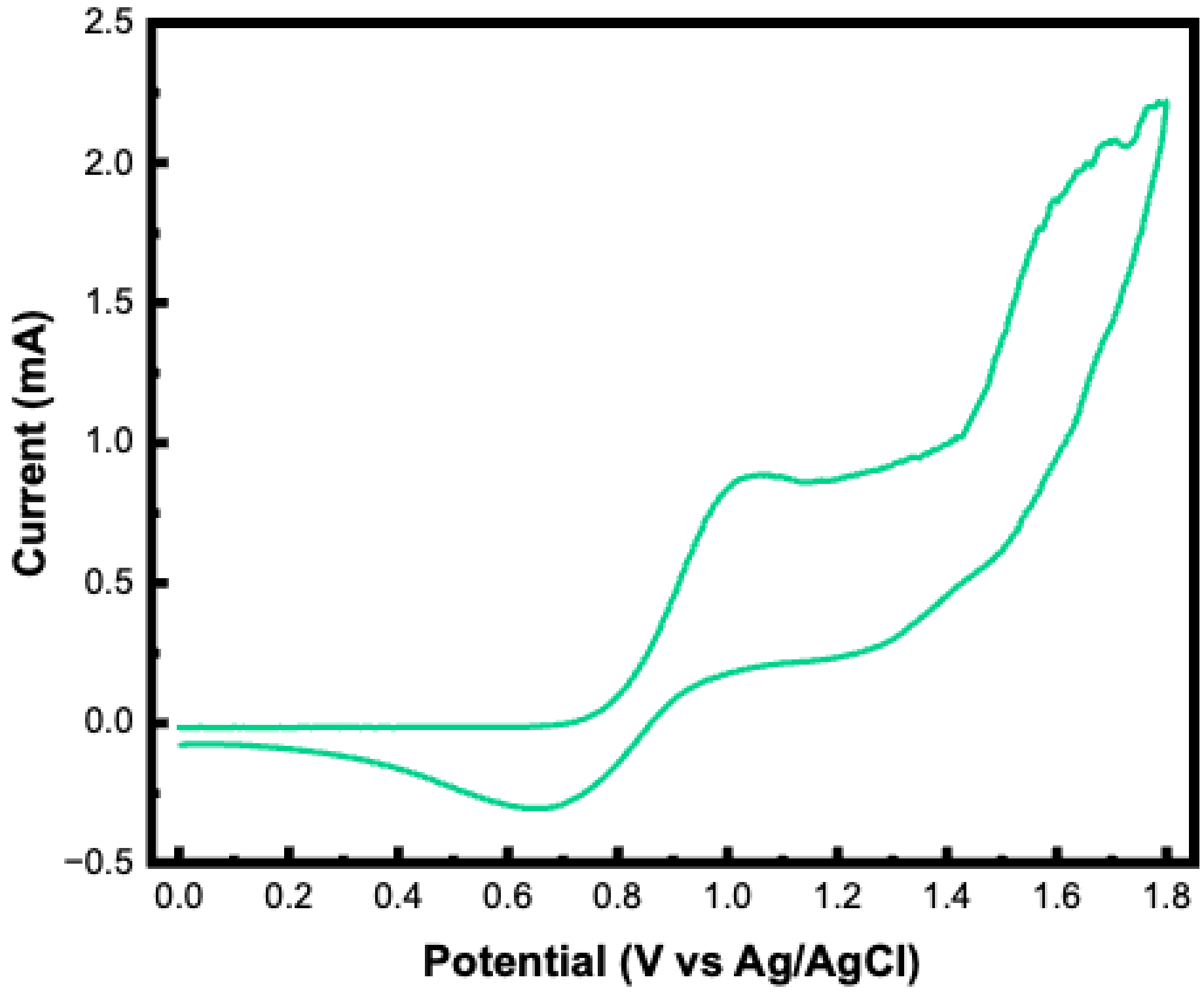

Figure S2. Corresponding Cyclic Voltammogram for Figure 1B. In this experiment, ECL microscopy of a *ca.* 2 µL aqueous droplet composed of 100 mM sodium oxalate and 100 mM KCl on an ITO working electrode was performed. The droplet was immersed in a bulk phase of 6 mM rubrene and 100 mM TBAP within 1,2-DCE. As shown above, to perform the ECL microscopy, cyclic voltammetry was carried out, sweeping from 0 to 1.8 V vs Ag/AgCl/1 M KCl at a scan rate of 50 mV/s.

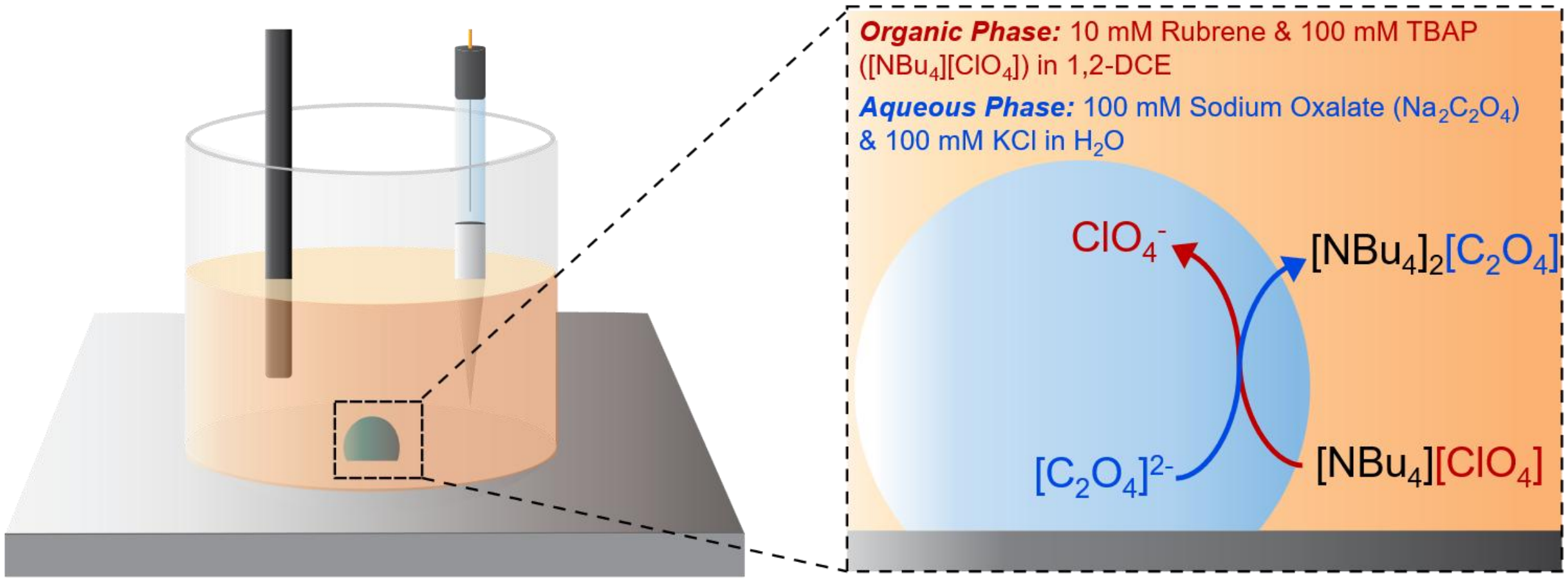

Figure S3. Proposed Mechanism of Oxalate Phase Transfer. In this phase transfer mechanism, oxalate anions ($[C_2O_4]^{2-}$) that are loaded into the aqueous droplet interact with tetrabutylammonium cations (NBu_4^+) that are present in high (100 mM) concentration in the bulk, organic phase. As NBu_4^+ is a well-known phase transfer agent that has previously been shown to drive the movement of anions between organic and aqueous phases,[1] in this model, it is postulated that NBu_4^+ facilitates the movement of $[C_2O_4]^{2-}$ from the aqueous droplet phase to the organic bulk phase. Then, to maintain electroneutrality, the anion of the supporting electrolyte, which, in this example, is perchlorate (ClO_4^-), partitions into the aqueous droplet phase. As ECL emission was observed in the bulk, organic phase, as shown in the ECL micrographs in Figure 1B, this model presents a hypothesis as to how $[C_2O_4]^{2-}$ enters the organic phase to enable this emission to occur. To further probe this phase transfer mechanism model, other organic supporting electrolytes with different anions were tested in Figure S4.

Reference:

1) Colón-Quintana, G. S.; Clarke, T. B.; Dick, J. E. Interfacial Solute Flux Promotes Emulsification at the Water|oil Interface. *Nat Commun* 2023, *14* (1), 705. https://doi.org/10.1038/s41467-023-35964-9.

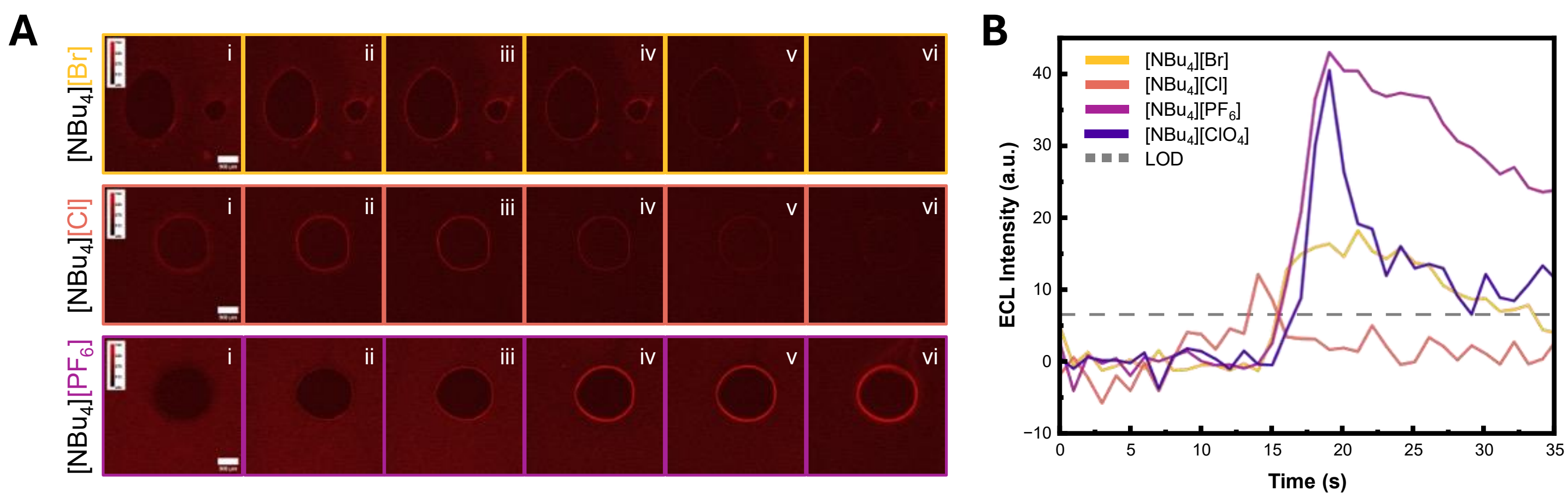

Figure S4. ECL Microscopy with Different Organic Phase Supporting Electrolytes. To test the phase transfer model presented in Figure S3, which describes the observed ECL emission in the bulk organic phase (Figure 1B), four organic supporting electrolytes were loaded into the organic phase. Each electrolyte contained the same tetrabutylammonium cation (NBu_4^+) and a different anion (Br^-, Cl^-, ClO_4^-, and PF_6^-). Hence, the examined organic supporting electrolytes were: $[NBu_4][Br]$, $[NBu_4][Cl]$, $[NBu_4][ClO_4]$, and $[NBu_4][PF_6]$. The experimental set-up consisted of a 2 μL aqueous droplet composed of 100 mM oxalate and 100 mM KCl within a bulk organic phase of 10 mM rubrene and 100 mM of the organic supporting electrolyte in 1,2-DCE. For this series of experiments, an ITO opto-electrochemical cell was used with an ITO working electrode, a GC rod counter electrode, and an Ag/AgCl/1 M KCl reference electrode connected to the system via an agarose salt bridge. **(A)** ECL microscopy of the different organic supporting electrolyte systems. In the top row (yellow), 100 mM of $[NBu_4][Br]$ supporting electrolyte was included in the bulk, organic phase. To drive the ECL microscopy, cyclic voltammetry was performed, sweeping from 0 V to 1.8 V at 50 mV/s. The ECL micrographs of the $[NBu_4][Br]$ supporting electrolyte system were taken at the following potentials: (i) 1.15 V, (ii) 1.40 V, (iii) 1.55 V, (iv) 1.75 V, (v) 1.65 V (back sweep), and (vi) 1.39 V (backsweep). In the middle row (orange), 100 mM of $[NBu_4][Cl]$ supporting electrolyte was included in the bulk, organic phase. To carry out the ECL microscopy, an amperometric i-t curve was employed, with 1.5 V vs Ag/AgCl/1 M KCl held for 120 s. The ECL micrographs of the $[NBu_4][Cl]$ supporting electrolyte system were taken at the following times: (i) 16.9 s, (ii) 17.9 s, (iii) 19.9 s, (iv) 22.9 s, (v) 24.9 s, and (vi) 26.9 s. In the bottom row (purple), 100 mM of $[NBu_4][Br]$ supporting electrolyte was included in the bulk, organic phase. To drive the ECL microscopy, cyclic voltammetry was performed, sweeping from 0 V to 1.8 V at 50 mV/s. The ECL micrographs of the $[NBu_4][PF_6]$ supporting electrolyte system were taken at the following potentials: (i) 1.12 V, (ii) 1.22 V, (iii) 1.32 V, (iv) 1.73 V, (v) 1.67 V (back sweep), and (vi) 0.00 V (backsweep). (B) ECL intensity vs. time overlay plot for the different organic supporting electrolytes. The plot shows the background ECL intensity of the bulk organic phase over time, measured via averaging the ECL intensity over a square ROI in the upper left corner of each of the different ECL microscopy trials shown in (A) and in Figure 1B for $[NBu_4][ClO_4]$. As can be seen, $[NBu_4][ClO_4]$ and $[NBu_4][PF_6]$ had the highest background ECL intensity, indicating that more oxalate partitions into the organic phase in those trials. For all trials, an EMCCD camera with a 4x objective and a 1x zoom was used with the following parameters: Gain 2, 10 MHz readout speed, 1-second exposure, and an EM gain of 400.

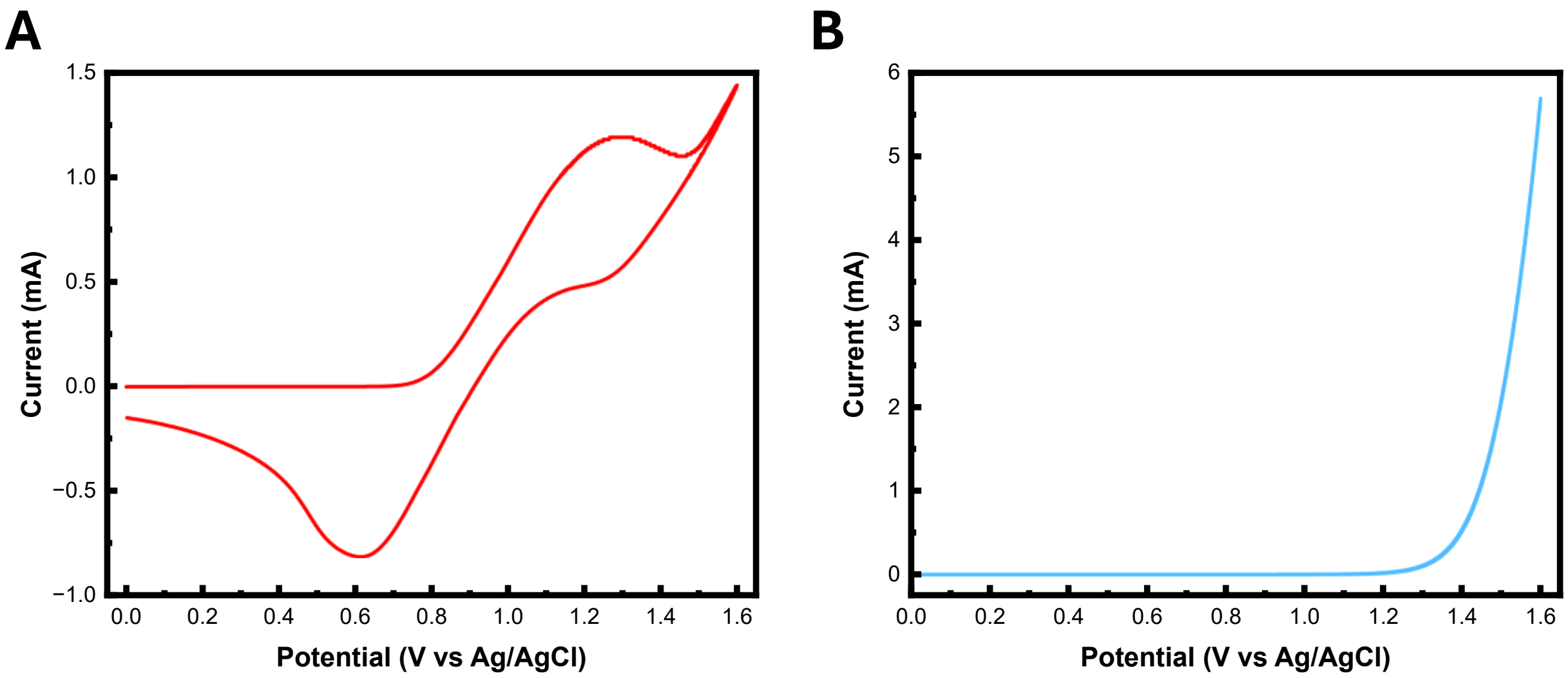

Figure S5. Cyclic Voltammetry of Rubrene and Oxalate Bulk Solutions. A) CV of 10 mM rubrene and 100 mM TBAP in 1,2-DCE. The CV was swept from 0 to 1.6 V vs Ag/AgCl and back at a scan rate of 50 mV/s. **B)** CV of 100 mM oxalate and 100 mM KCl in water. The CV was swept from 0 to 1.6 V vs Ag/AgCl and back at a scan rate of 50 mV/s. Both CVs were collected with an ITO working electrode, glassy carbon rod counter electrode, and an Ag/AgCl/1 M KCl reference electrode connected to the ITO cell via an agarose salt bridge.

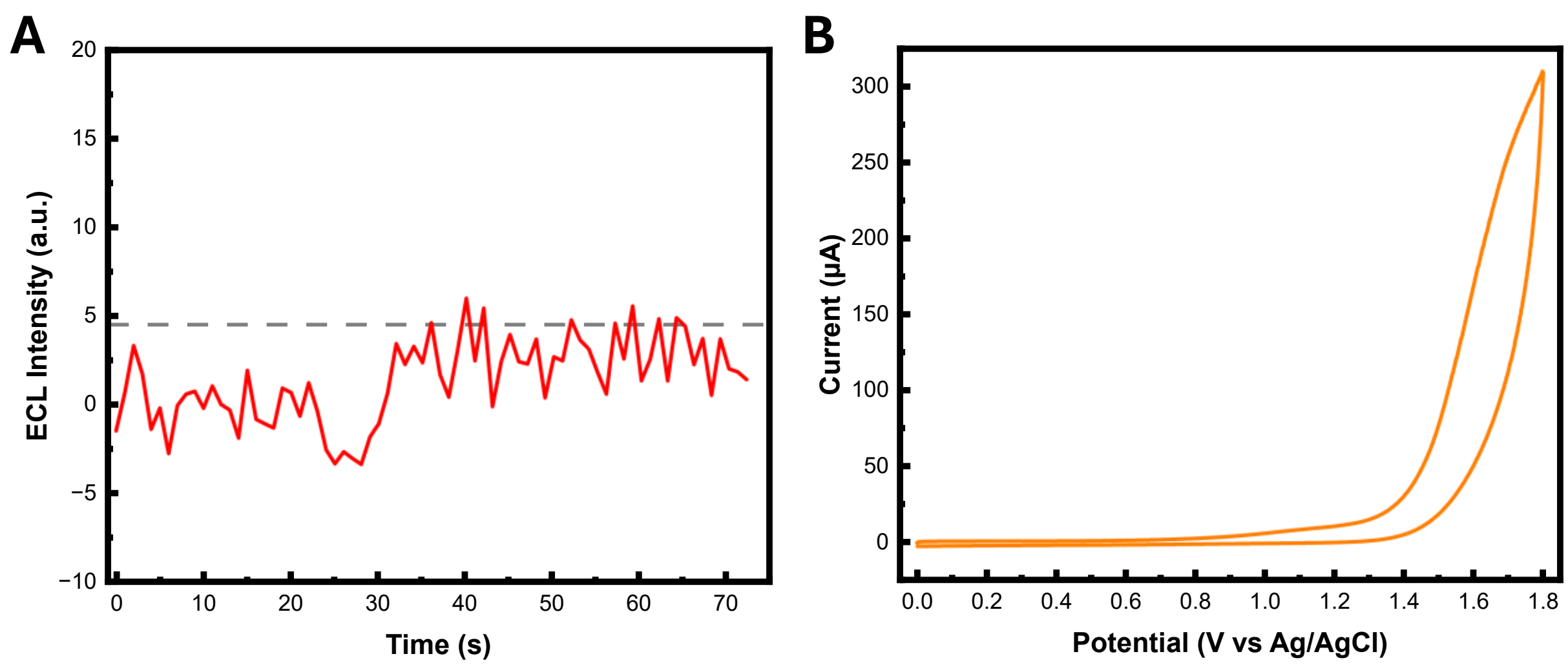

Figure S6. Control: ECL Microscopy and Cyclic Voltammetry without Rubrene. In this experiment, ECL microscopy was performed on a *ca.* 2 µL aqueous droplet composed of 100 mM sodium oxalate and 100 mM KCl on an ITO working electrode. The droplet was immersed in a bulk phase of 100 mM TBAP within 1,2-DCE. **(A)** ECL intensity vs time plot demonstrating that no sustained ECL emission was observed above the LOD (dark grey, dashed line) for this system. Hence, for this ECL system, rubrene is required for ECL emission. For the ECL microscopy, an EMCCD camera with a 4x objective and a 1x zoom was used with the following parameters: Gain 2, 10 MHz readout speed, 1 second exposure, and an EM gain of 400. **(B)** To perform the ECL microscopy, cyclic voltammetry was carried out, sweeping from 0 to 1.8 V vs Ag/AgCl/1 M KCl at a scan rate of 50 mV/s.

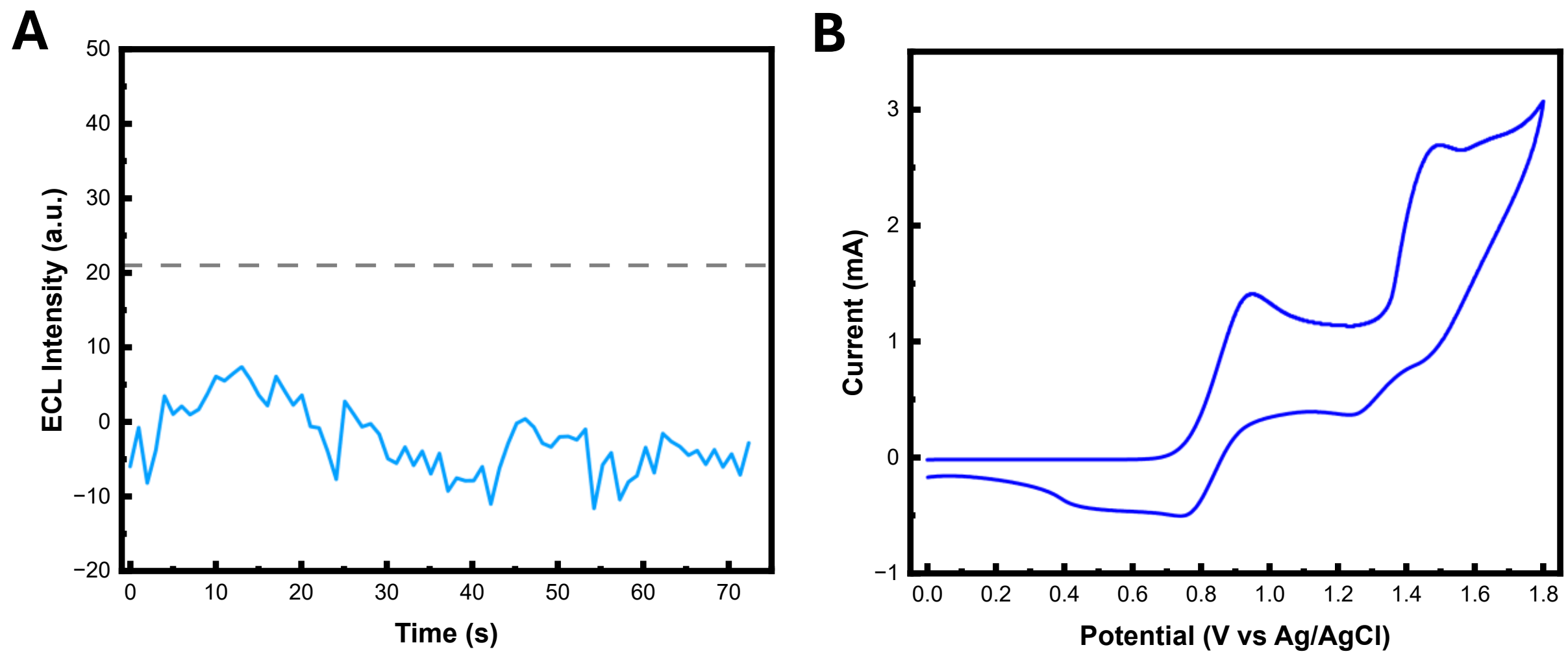

Figure S7. Control: Cyclic Voltammetry and ECL Microscopy without Oxalate. In this experiment, ECL microscopy was performed on a *ca.* 2 µL aqueous droplet composed of 100 mM KCl only on an ITO working electrode. The droplet was immersed in a bulk phase of 10 mM rubrene and 100 mM TBAP within 1,2-DCE. **(A)** ECL intensity vs time plot demonstrating that no ECL signal was detected above the LOD (dark grey, dashed line) for this system. Hence, for this ECL system, oxalate is required for ECL emission. For the ECL microscopy, an EMCCD camera with a 4x objective and a 1x zoom was used with the following parameters: Gain 2, 10 MHz readout speed, 1 second exposure, and an EM gain of 400. **(B)** To perform the ECL microscopy, cyclic voltammetry was carried out, sweeping from 0 to 1.8 V vs Ag/AgCl/1 M KCl at a scan rate of 50 mV/s.

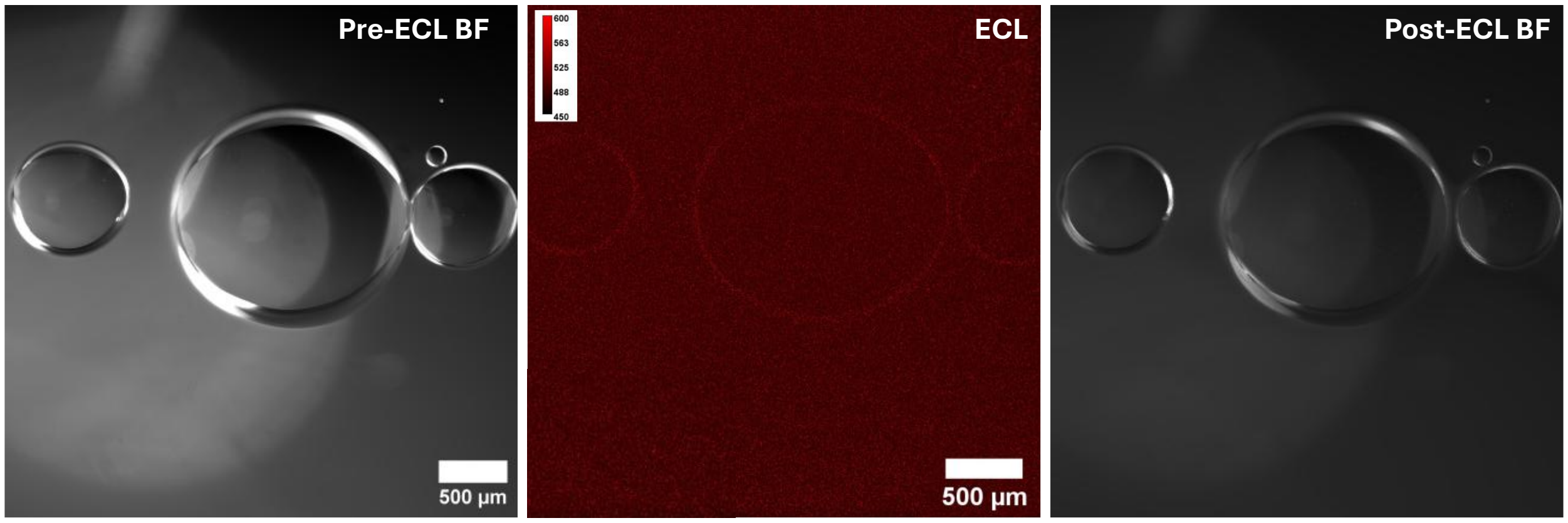

Figure S8. BF and ECL Microscopy of Different-Sized Droplets. The aqueous droplets were composed of 100 mM oxalate and 100 mM KCl, and were immersed in a bulk solution of 10 mM rubrene and 100 mM TBAP in 1,2-DCE. The volume of the large, central droplet was ~ 1 µL, while the smaller droplets on either side had a volume of ~ 0.5 µL. To carry out the ECL microscopy, a CV was swept from 0 to 1.8 V vs Ag/AgCl and back at a scan rate of 50 mV/s. The system consisted of an ITO working electrode, a glassy carbon rod counter electrode, and an Ag/AgCl/1 M KCl reference electrode connected to the ITO cell via an agarose salt bridge. BF micrographs were collected before and after carrying out the ECL microscopy, with the ECL micrograph shown being the highest ECL intensity frame. No droplet reorganization was observed.

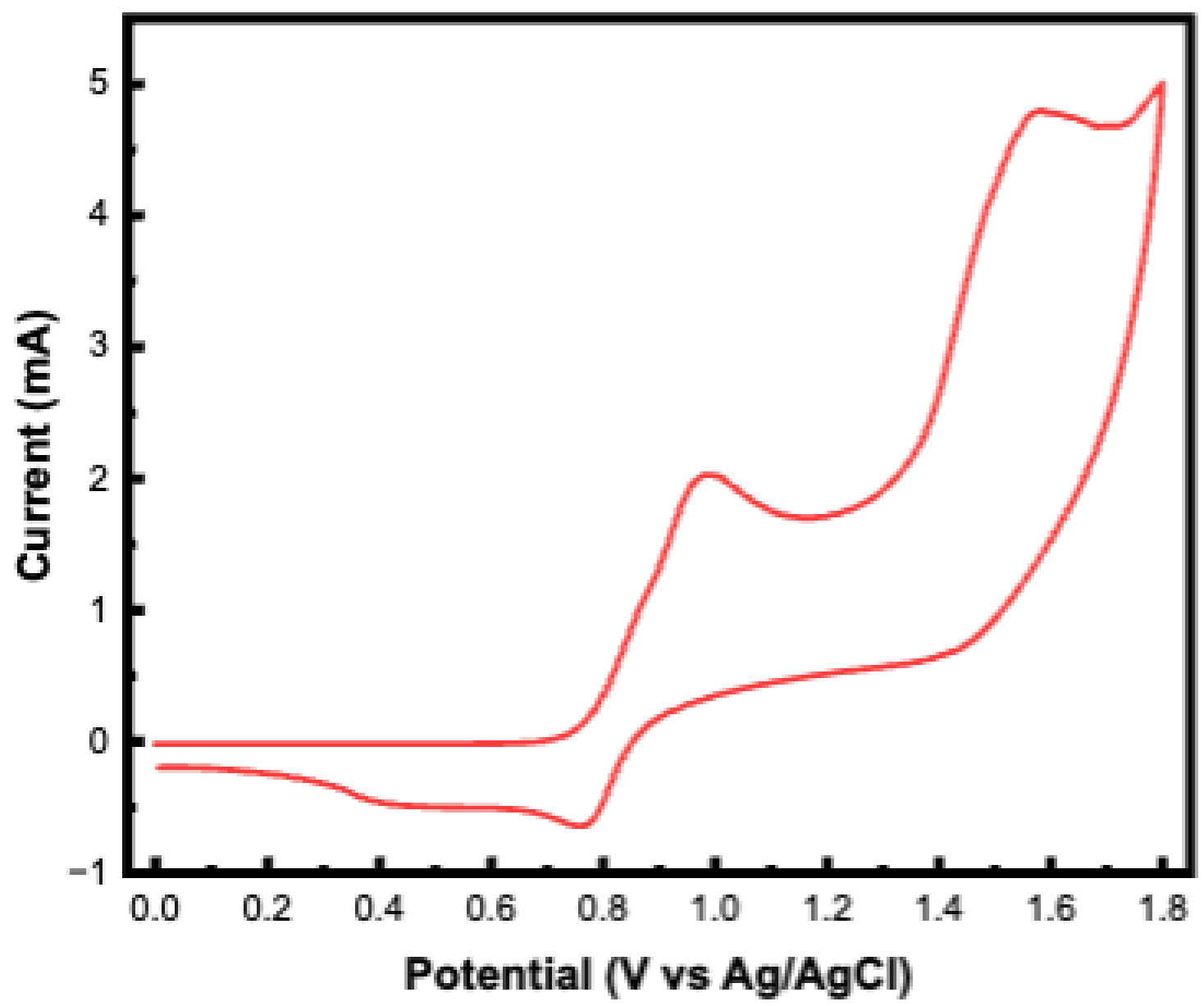

Figure S9. Correlated Cyclic Voltammogram for Figure 2B and Figure 2C. In this experiment, ECL microscopy of a *ca.* 2 µL aqueous droplet composed of 80 mM sodium oxalate and 100 mM KCl on an ITO working electrode was performed. The droplet was immersed in a bulk phase of 10 mM rubrene and 100 mM TBAP within 1,2-DCE. As shown above, to perform the ECL microscopy, cyclic voltammetry was carried out, sweeping from 0 to 1.8 V vs Ag/AgCl/1 M KCl at a scan rate of 50 mV/s.

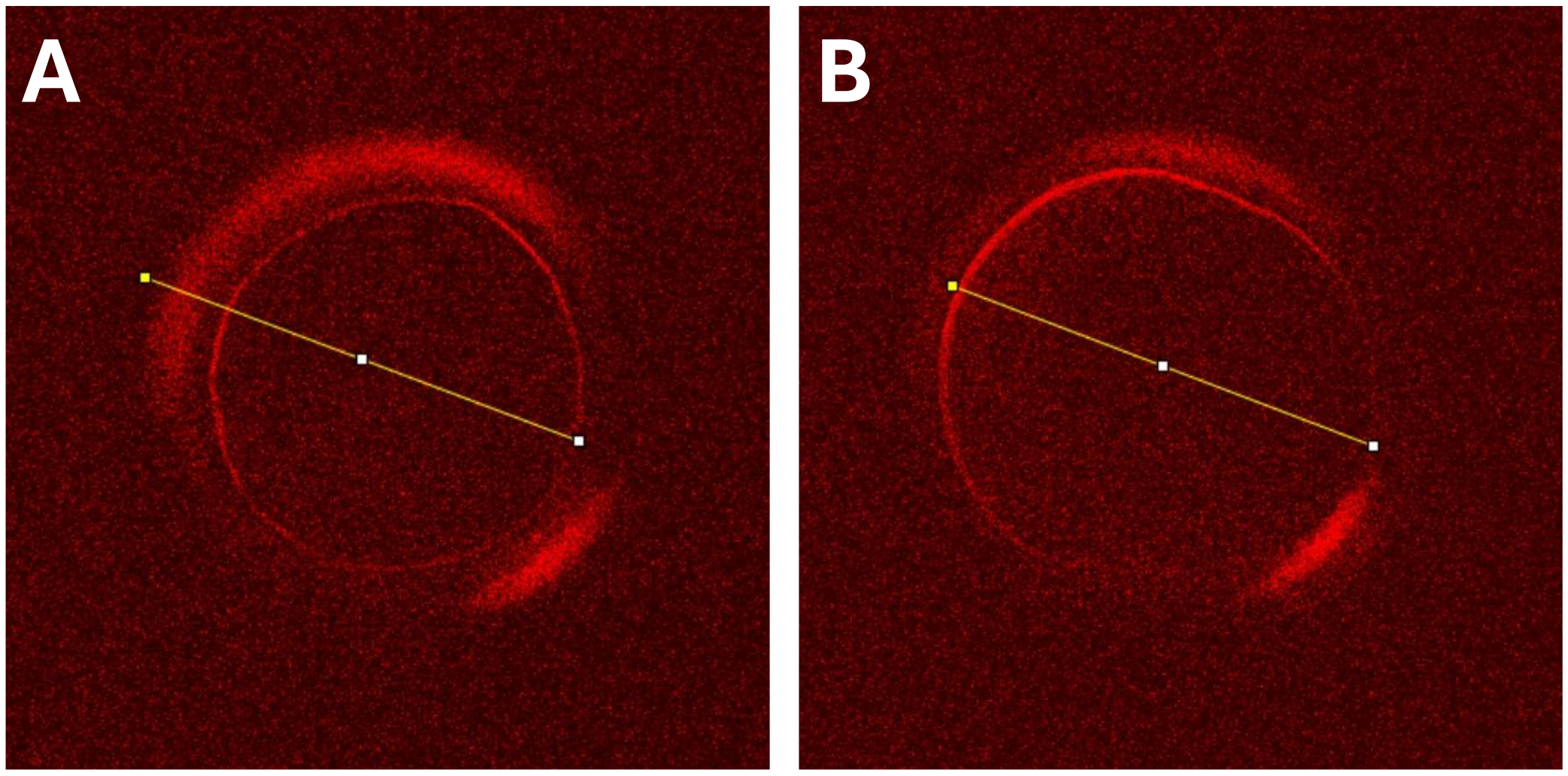

Figure S10. Line used for the Line Scans in Figure 2C. The same line (shown in yellow) that was drawn across the droplet was used for both the line scans. **(A)** Shows the line across the droplet before the reorganization event, while **(B)** shows the line across the droplet after the reorganization event.

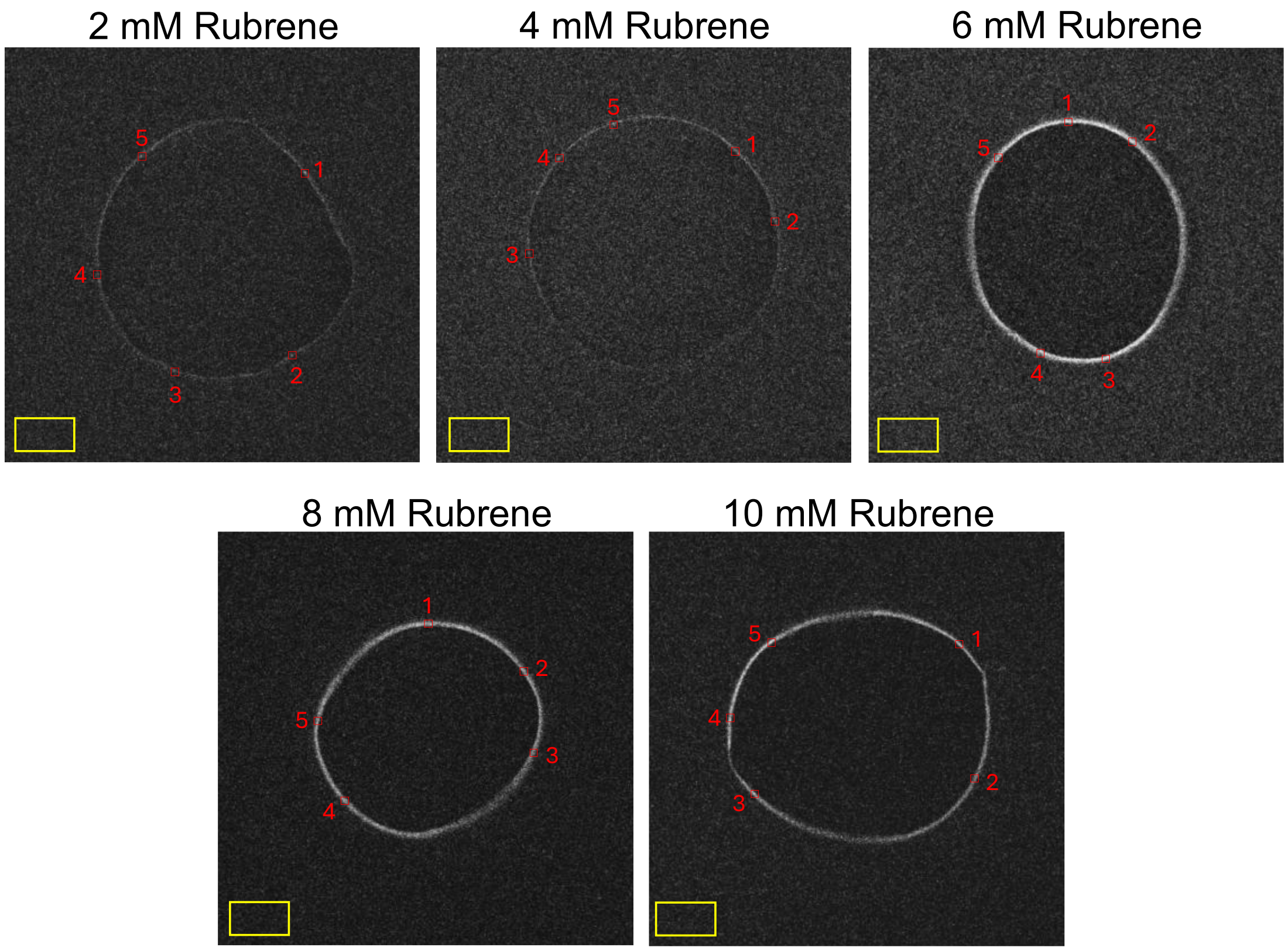

Figure S11. Selected ROIs for ECL Intensity Measurements at Different Rubrene Concentrations. For each experiment, ECL microscopy of a *ca.* 2 µL aqueous droplet composed of 100 mM sodium oxalate and 100 mM KCl on an ITO working electrode was performed. The droplet was immersed in a bulk phase of rubrene at different concentrations and 100 mM TBAP within 1,2-DCE. To perform the ECL microscopy, cyclic voltammetry was carried out, sweeping from 0 to 1.8 V vs Ag/AgCl/1 M KCl at a scan rate of 50 mV/s. The different concentrations of rubrene tested were: 2 mM, 4 mM, 6 mM, 8 mM, and 10 mM. To determine the average ECL intensity for each of the different rubrene concentrations for Figure 2D, five ROIs were selected along the triphasic boundary where ECL emission occurred. Each of the selected ROIs is shown for every rubrene concentration, evidenced by red squares and labelled with the red numbers. For the background subtraction, a square ROI (yellow) in the bottom left corner was used. For the ECL microscopy, an EMCCD camera with a 4x objective and a 1x zoom was used with the following parameters: Gain 2, 10 MHz readout speed, 1 second exposure, and an EM gain of 400.

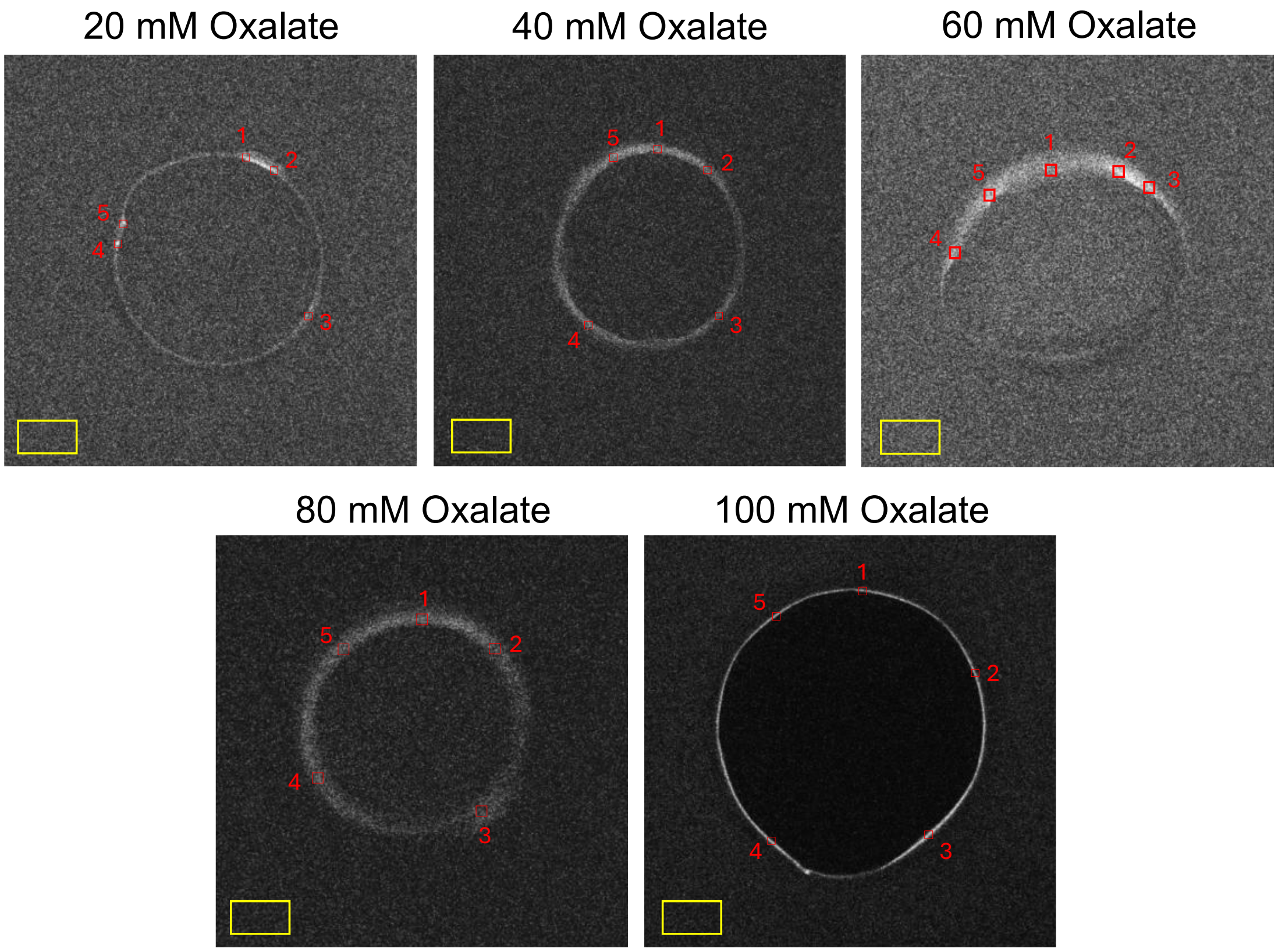

Figure S12. Selected ROIs for ECL Intensity Measurements at Different Oxalate Concentrations. For each experiment, ECL microscopy of a *ca.* 2 µL aqueous droplet composed of sodium oxalate at different concentrations and 100 mM KCl on an ITO working electrode was performed. The droplet was immersed in a bulk phase of 10 mM rubrene and 100 mM TBAP within 1,2-DCE. To perform the ECL microscopy, cyclic voltammetry was carried out, sweeping from 0 to 1.8 V vs Ag/AgCl/1 M KCl at a scan rate of 50 mV/s. The different concentrations of oxalate tested were: 20 mM, 40 mM, 60 mM, 80 mM, and 100 mM. To determine the average ECL intensity for each of the different rubrene concentrations for Figure 2E, five ROIs were selected along the triphasic boundary where ECL emission occurred. Each of the selected ROIs is shown for every oxalate concentration, evidenced by red squares and labelled with the red numbers. For the background subtraction, a square ROI (yellow) in the bottom left corner was used. For the ECL microscopy, an EMCCD camera with a 4x objective and a 1x zoom was used with the following parameters: Gain 2, 10 MHz readout speed, 1 second exposure, and an EM gain of 400.

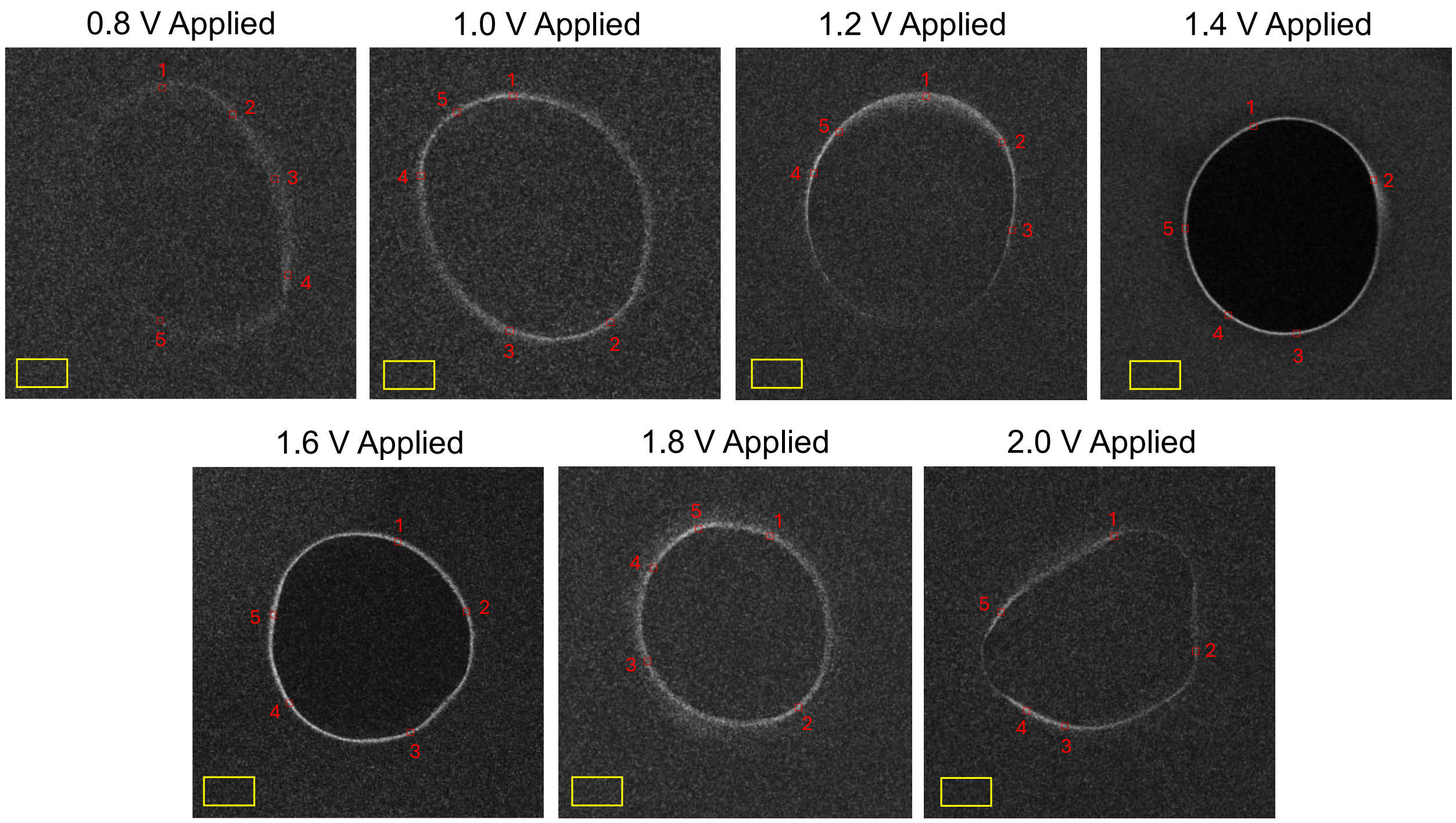

Figure S13. Selected ROIs for ECL Intensity Measurements at Different Applied Potentials. For each experiment, ECL microscopy was performed on a *ca.* 2 µL aqueous droplet composed of 100 mM sodium oxalate and 100 mM KCl, using an ITO working electrode. The droplet was immersed in a bulk phase of 10 mM rubrene and 100 mM TBAP within 1,2-DCE. To perform the ECL microscopy, amperometric i-t curves were used, with varying potentials held for 60 s. The different applied potentials tested were: 0.8 V, 1.0 V, 1.2 V, 1.4 V, 1.6 V, 1.8 V, and 2.0 V vs Ag/AgCl/1 M KCl. To determine the average ECL intensity for each of the different rubrene concentrations for Figure 2F, five ROIs were selected along the triphasic boundary where ECL emission occurred. Each of the selected ROIs is shown for every different applied potential, evidenced by red squares and labelled with the red numbers. For the background subtraction, a square ROI (yellow) in the bottom left corner was used. For the ECL microscopy, an EMCCD camera with a 4x objective and a 1x zoom was used with the following parameters: Gain 2, 10 MHz readout speed, 1 second exposure, and an EM gain of 400.

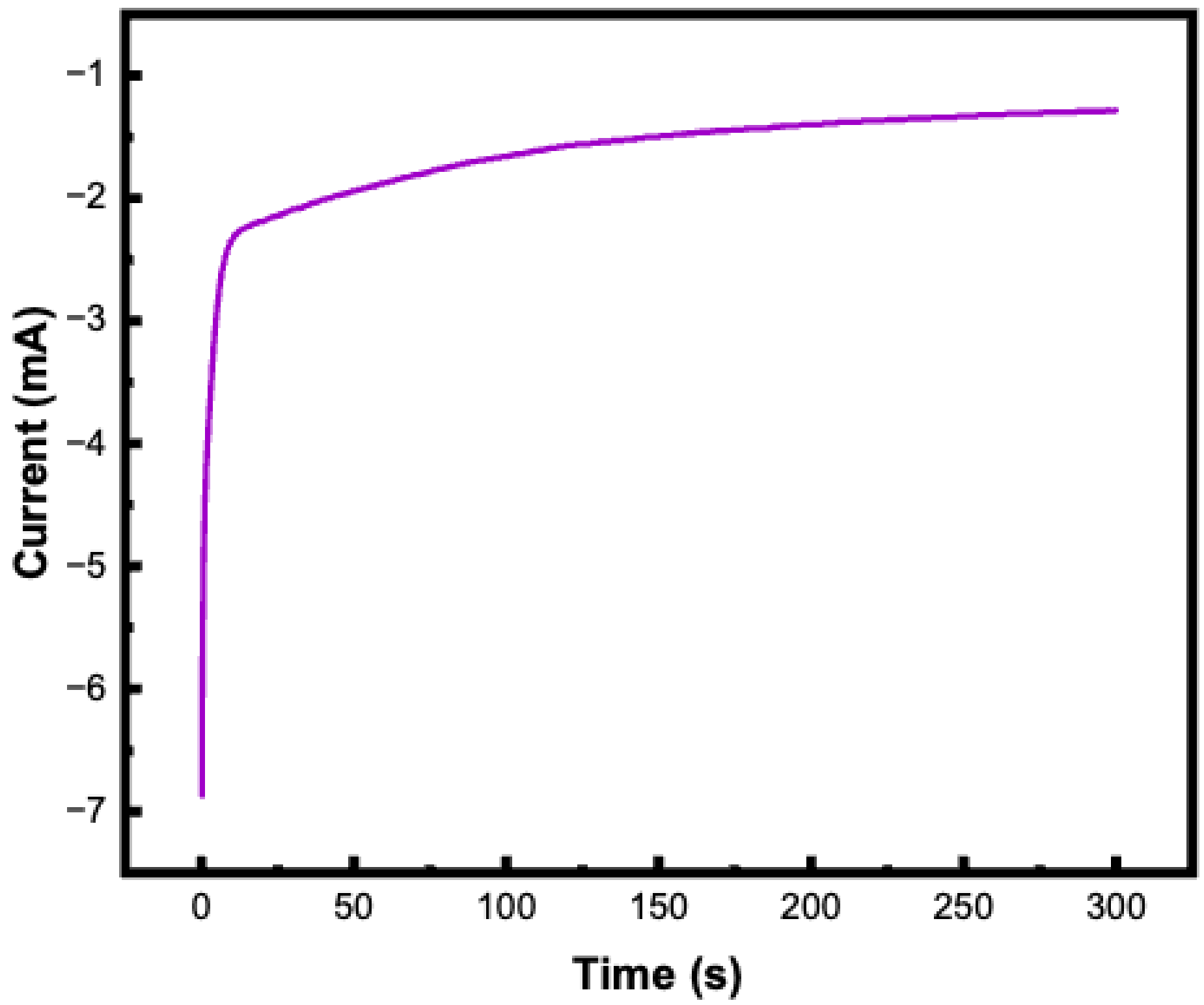

Figure S14. Amperometric i-t curve corresponding to Figure 3. In this experiment, ECL microscopy of an emulsion composed of *ca.* 100 µL of an organic, droplet phase composed of 10 mM rubrene and 100 mM TBAP within 1,2-DCE and 5 mL of an aqueous, continuous phase consisting of 100 mM sodium oxalate and 100 mM KCl was interrogated. As shown above, to perform the ECL microscopy, an amperometric i-t curve held for 300 s at 1.6 V vs Ag/AgCl/1 M KCl was carried out.

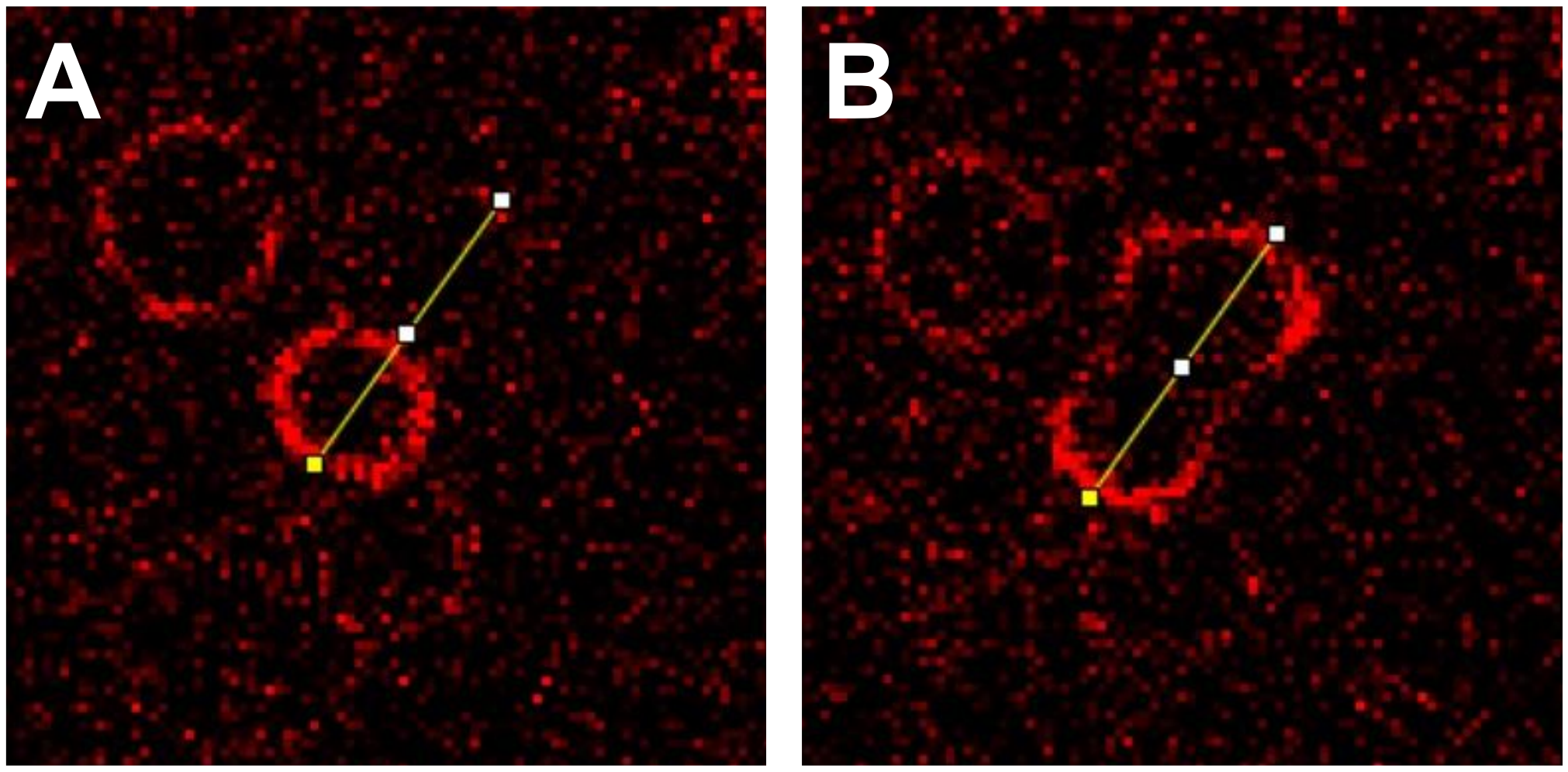

Figure S15. Line used for Figure 3C line scans. The same line (shown in yellow) that was drawn across the droplet was used for both the line scans. **(A)** Shows the line across the droplet in the ECL micrograph captured at 75.07 s, used for the line scan (orange) shown in Figure 3C. **(B)** Shows the line across the droplet in the ECL micrograph captured at 85.08 s, used for the line scan (red) shown in Figure 3C.

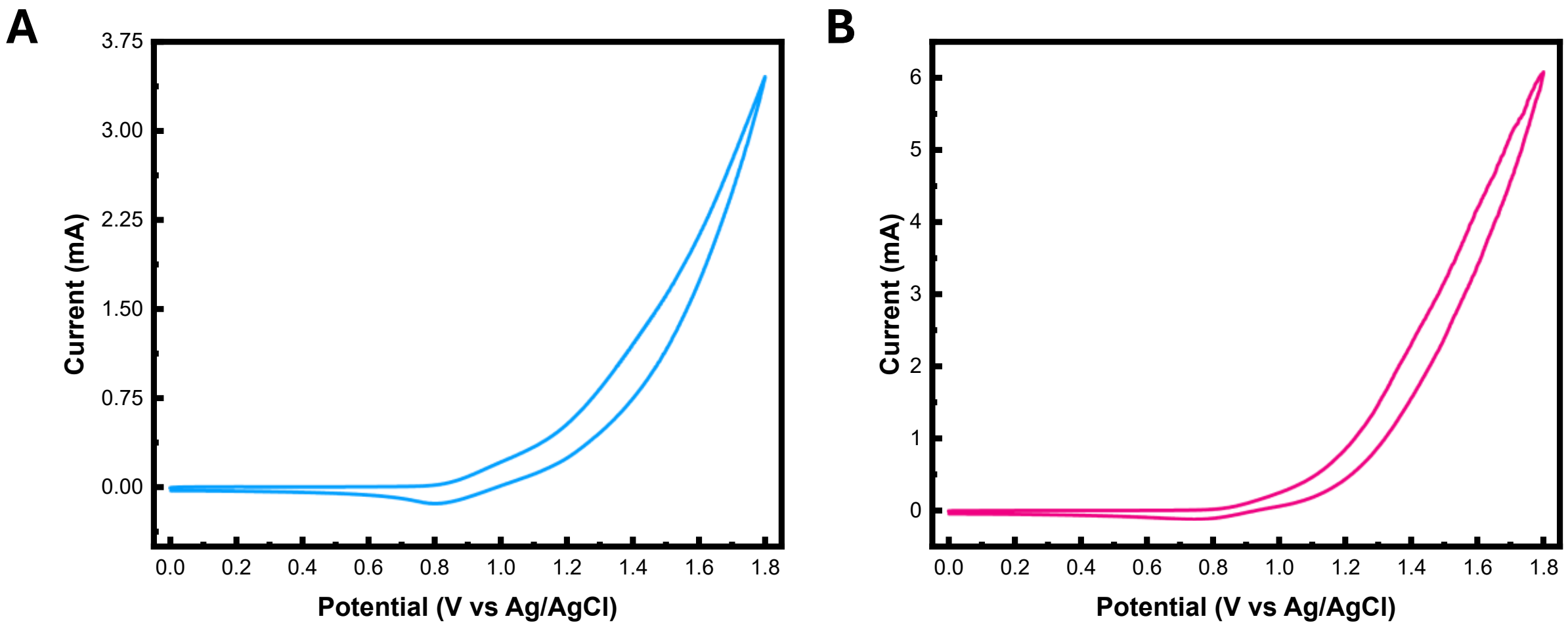

Figure S16. Cyclic Voltammograms corresponding to Figures 4B, 4C, and 4D. In this experiment, ECL microscopy of a bulk liquid|bulk liquid|electrode system was carried out, with Figure 4C focused at the triphasic boundary, while Figure 4D is focused on the end of the working electrode. The bottom, organic phase was composed of 10 mM rubrene and 100 mM TBAP in 1,2-DCE, while the top, aqueous phase consisted of 100 mM sodium oxalate and 100 mM KCl. Approximately 2 mL of each phase was added to the glass opto-electrochemical cell, and a GC rod working electrode was placed into the system so that it penetrated both phases, with the end of the rod immersed in the bulk, organic phase. As shown above, to drive the ECL reaction, cyclic voltammetry was performed, sweeping from 0 to 1.8 V vs Ag/AgCl/1 M KCl at a scan rate of 50 mV/s. **(A)** shows the CV corresponding to the ECL micrographs in Figure 4C, while **(B)** shows the CV corresponding to the ECL micrographs in Figure 4D.

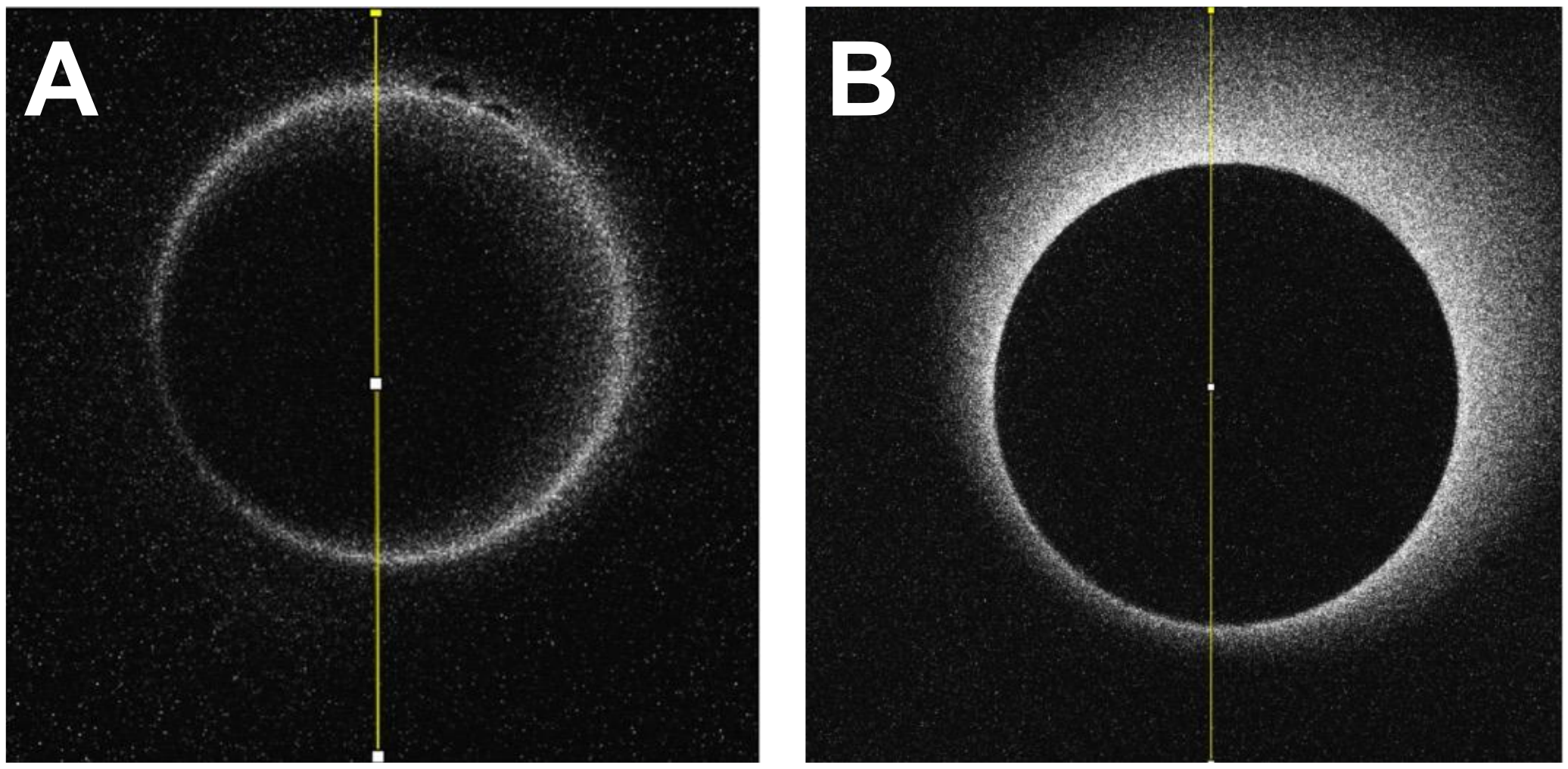

Figure S17. Lines used for the Line Scans in Figure 4B. (A) Shows the line across the ECL micrograph in Figure 4C captured at 1.10 V vs Ag/AgCl/1 M KCl used for the line scan (red) shown in Figure 4B. **(B)** Shows the line across the ECL micrograph in Figure 4D captured at 1.22 V vs Ag/AgCl/1 M KCl used for the line scan (blue) shown in Figure 4B.

SI Movie Descriptions

Movie S1 (separate file). Corresponds to the main text *Figure 1B*. ECL microscopy of a *ca.* 2 μL aqueous droplet composed of 100 mM sodium oxalate and 100 mM KCl on an ITO working electrode. The droplet was immersed in a bulk phase of 6 mM rubrene and 100 mM TBAP within 1,2-DCE. Cyclic voltammetry was performed, sweeping from 0 to 1.8 V vs Ag/AgCl/1 M KCl at a scan rate of 50 mV/s. The scale bar is 500 μm.

Movie S2 (separate file). Corresponds to the main text *Figure 1C*. In other words, Movie S2 shows the ECL microscopy trial used in the correlated ECL intensity and cyclic voltammetry plot. The system consisted of a *ca.* 2 μL aqueous droplet composed of 100 mM sodium oxalate and 100 mM KCl on an ITO working electrode. The droplet was immersed in a bulk phase of 10 mM rubrene and 100 mM TBAP within 1,2-DCE. Cyclic voltammetry was performed, sweeping from 0 to 1.8 V vs Ag/AgCl/1 M KCl at a scan rate of 50 mV/s. The scale bar is 500 μm.

Movie S3 (separate file). Corresponds to the main text *Figure 2B* and *Figure 2C*. ECL microscopy of a *ca.* 2 μL aqueous droplet composed of 80 mM sodium oxalate and 100 mM KCl on an ITO working electrode. The droplet was immersed in a bulk phase of 10 mM rubrene and 100 mM TBAP within 1,2-DCE. Cyclic voltammetry was performed, sweeping from 0 to 1.8 V vs Ag/AgCl/1 M KCl at a scan rate of 50 mV/s. The scale bar is 500 μm.

Movie S4 (separate file). Corresponds to the main text *Figure 3B* and *Figure 3C*. ECL microscopy of an emulsion system. The droplet phase, the organic phase, was composed of 10 mM rubrene and 100 mM TBAP in 1,2-DCE. The continuous (or bulk) phase, the aqueous phase, consisted of 100 mM sodium oxalate and 100 mM KCl. To prepare the emulsion, 100 μL of the organic droplet phase was added to 5 mL of the aqueous continuous phase, and the solution was then sonicated and left to settle on the ITO working electrode. An amperometric i-t curve was performed, where 1.6 V vs Ag/AgCl/1 M KCl was held for 300 s. The scale bar is 500 μm

Movie S5 (separate file). Corresponds to the main text *Figure 4C*. ECL microscopy of a bulk liquid|bulk liquid|electrode triphasic boundary with the focus of the camera on the triphasic boundary. The bottom, organic phase was composed of 10 mM rubrene and 100 mM TBAP in 1,2-DCE, while the top, aqueous phase consisted of 100 mM sodium oxalate and 100 mM KCl. Approximately 2 mL of each phase was added to the glass opto-electrochemical cell, and a GC rod working electrode was placed into the system so that it penetrated both phases, with the end of the rod immersed in the bulk, organic phase. Cyclic voltammetry was performed, sweeping from 0 to 1.8 V vs Ag/AgCl/1 M KCl at a scan rate of 50 mV/s. The scale bar is 500 μm.

Movie S6 (separate file). Corresponds to the main text *Figure 4D*. ECL microscopy of a bulk liquid|bulk liquid|electrode triphasic boundary with the focus of the camera at the end of the GC rod electrode. The bottom, organic phase was composed of 10 mM rubrene and 100 mM TBAP in 1,2-DCE, while the top, aqueous phase consisted of 100 mM sodium oxalate and 100 mM KCl. Approximately 2 mL of each phase was added to the glass opto-electrochemical cell, and a GC rod working electrode was placed into the system so that it penetrated both phases, with the end of the rod immersed in the bulk, organic phase. Cyclic voltammetry was performed, sweeping from 0 to 1.8 V vs Ag/AgCl/1 M KCl at a scan rate of 50 mV/s. The scale bar is 500 μm.
